# Selective PAC1 Receptor Antagonist, PA-810-04 Attenuates Oxaliplatin-Induced Hyperexcitability and Coordinated Firing in Cultured Dorsal Root Ganglion Neurons

**DOI:** 10.3390/ijms27219665

**Published:** 2026-10-28

**Authors:** Taiyo Arima, Kazuhiro Takahama, Kimitaka Suetsugu, Yukiko Kobae, Haruki Iwai, Ichiro Takasaki, Takashi Kurihara, Hideyuki Terazono

**Affiliations:** 1 Department of Clinical Pharmacy and Pharmacology, Graduate School of Medical and Dental Sciences, Kagoshima University, 8-35-1 Sakuragaoka, Kagoshima 890-8544, Japan; ta126245@m.kufm.kagoshima-u.ac.jp (T.A.); ksuetsugu@kufm.kagoshima-u.ac.jp (K.S.); ykobae@m.kufm.kagoshima-u.ac.jp (Y.K.); 2 Department of Pharmacy, Kagoshima University Hospital, 8-35-1 Sakuragaoka, Kagoshima 890-8520, Japan; takahama@kufm.kagoshima-u.ac.jp; 3 Department of Oral Anatomy and Cell Biology, Graduate School of Medical and Dental Sciences, Kagoshima University, 8-35-1 Sakuragaoka, Kagoshima 890-8544, Japan; haruki@dent.kagoshima-u.ac.jp; 4 Laboratory of Pharmacology, Graduate School of Pharma-Medical Sciences, University of Toyama, 3190 Gofuku, Toyama 930-8555, Japan; takasaki@eng.u-toyama.ac.jp; 5 Department of Pharmacology, Graduate School of Medical and Dental Sciences, Kagoshima University, 8-35-1 Sakuragaoka, Kagoshima 890-8544, Japan; tkmphm10@m.kufm.kagoshima-u.ac.jp

**Keywords:** oxaliplatin-induced peripheral neuropathy, PACAP, PAC1 receptor, dorsal root ganglion, satellite glial cell, multielectrode array

## Abstract

Oxaliplatin-induced peripheral neuropathy (OIPN) is a major dose-limiting adverse effect of chemotherapy, and dorsal root ganglion (DRG) neurons are its principal targets. Although pituitary adenylate cyclase-activating polypeptide (PACAP)/PAC1 receptor signaling has been implicated in neuropathic pain, its contribution to the oxaliplatin-induced hyperexcitability of primary sensory neurons remains unknown. We used a dual-axis multi-electrode array (MEA) platform quantifying the total spike count (TSC) and synchronous spike ratio (SSR), indices of overall activity and coordinated firing. Short-term oxaliplatin exposure produced concentration-dependent increases in TSC and SSR in cultured DRG neurons, which were significantly attenuated by the selective PAC1 receptor antagonist, PA-810-04. Similarly, conditioned medium from oxaliplatin-treated satellite glial cell (SGC)-enriched cultures increased TSC and SSR in naïve DRG neurons, and these effects were suppressed by PA-810-04. Exogenous PACAP alone increased TSC concentration-dependently, with only a modest effect on SSR, as a positive control. These findings identify a PA-810-04-sensitive component of early oxaliplatin-induced hyperexcitability in primary sensory neurons, while the proposed neuron–glia mechanism remains hypothetical. They also provide a quantitative in vitro approach for investigating neuron–glia interactions in early OIPN and evaluating PAC1-targeted candidates.

## 1. Introduction

Oxaliplatin (L-OHP) is a platinum-based chemotherapeutic agent that is widely used to treat colorectal and other gastrointestinal cancers [1]. However, L-OHP-induced peripheral neuropathy (OIPN) remains one of the most common dose-limiting adverse effects, and often requires dose reduction or discontinuation of chemotherapy. Acute OIPN develops within hours to days of treatment and is characterized by cold hypersensitivity, paresthesia, and spontaneous sensory disturbances, whereas chronic OIPN can persist long after completion of chemotherapy [2,3]. Despite its clinical importance, no established therapy has effectively prevented the development of OIPN, largely because the cellular and molecular mechanisms underlying its early pathogenesis remain unclear [2,4].

Primary sensory neurons in the dorsal root ganglion (DRG) are the principal cellular targets of L-OHP neurotoxicity. Therefore, elucidating the molecular signaling mechanisms operating at the primary sensory neuron level is of particular importance for understanding OIPN initiation [2,4]. Increasing evidence indicates that acute neuronal hyperexcitability develops before overt structural degeneration and represents a critical early event in the pathogenesis of OIPN. Because pathological changes in DRG neurons precede central sensitization, understanding the molecular events occurring in primary sensory neurons is essential for elucidating the initiation of OIPN.

In addition to neuronal dysfunction, satellite glial cells (SGCs), which tightly envelop the DRG neuronal somata, have emerged as important regulators of sensory neuron excitability [5]. Following peripheral nerve injury or inflammatory insults, activated SGCs release soluble mediators that modulate neuronal activity through bidirectional neuron–glial communication [5,6]. Although SGC activation following L-OHP treatment has been reported, the signaling mechanisms mediating SGC-dependent modulation of primary sensory neurons during the early phase of OIPN remain poorly understood [6].

Pituitary adenylate cyclase-activating polypeptide (PACAP) is a neuropeptide that regulates nociceptive transmission, neuroinflammation, and neuron–glial communication, primarily through PAC1 receptor [7,8]. Growing evidence indicates that PACAP/PAC1 receptor signaling contributes to central sensitization underlying the development of neuropathic pain [9,10,11,12,13,14]. In particular, we recently demonstrated that pharmacological inhibition of PAC1 receptors attenuates the spinal mechanisms underlying L-OHP-induced neuropathic pain, suggesting that spinal PACAP/PAC1 signaling contributes to OIPN pathogenesis [15]. This work, however, concerned spinal mechanisms assessed by pain behavior and did not interrogate the primary sensory neurons themselves, nor did it address how L-OHP reorganizes activity within a sensory neuron population. Two specific questions therefore remain unanswered: whether PACAP/PAC1 signaling contributes to the onset of OIPN by promoting the hyperexcitability of primary sensory neurons, the principal targets of L-OHP neurotoxicity, and whether this contribution extends beyond spike output to the temporal coordination of firing across the neuronal population. The present study addresses these questions by directly recording the firing activity of DRG neurons electrophysiologically rather than inferring neuronal activity from pain-related behaviors, by resolving the response into spike output and population-level coordinated firing, and by examining the contribution of soluble factors released from SGC-enriched cultures in an in vitro preparation designed to isolate the acute phase of OIPN. This integrated approach enables the analysis of neuronal activity and neuron–glia interactions under controlled conditions that are difficult to achieve in vivo.

To address this question, we employed a dual-axis multielectrode array (MEA) platform capable of simultaneously quantifying the total spike count (TSC), an index of overall neuronal activity, and synchronous spike ratio (SSR), a measure of coordinated neuronal firing [16,17,18]. Using cultured DRG neurons, exogenous PACAP stimulation, pharmacological inhibition of PAC1 receptors, and conditioned medium derived from L-OHP-treated SGC-enriched cultures, we investigated whether PACAP/PAC1 receptor signaling contributes to L-OHP-induced neuronal hyperexcitability in primary sensory neurons.

## 2. Results

### 2.1. Expression of PACAP and PAC1 Receptor in Cultured DRG Preparations

Reverse transcription polymerase chain reaction (RT-PCR) confirmed expression of both PACAP (*Adcyap1*) and the PAC1 receptor (*Adcyap1r1*) mRNAs in the neuron-enriched DRG cultures used in this study (Supplementary Figure S1A). Immunocytochemical analysis further demonstrated PACAP immunoreactivity in the cultured DRG neurons. Double immunofluorescence staining detected PACAP immunoreactivity in a large proportion of calcitonin gene-related peptide (CGRP)-immunoreactive neurons, indicating that PACAP was expressed in at least one subpopulation of peptidergic nociceptive sensory neurons under the present culture conditions (Supplementary Figure S1B). Together, these findings indicate that the cultured preparations expressed *Adcyap1* and *Adcyap1r1* transcripts and that PACAP peptide was present in neurons. However, because these cultures also contained non-neuronal cells, the cellular source of the detected *Adcyap1r1* transcripts could not be determined.

### 2.2. Exogenous PACAP Increased the Total Spike Count of Cultured DRG Neurons

DRG neuronal activity was quantified from MEA recordings using two complementary readouts (Section 4.7). The population firing-rate histogram displays the time course of the firing rate averaged across the 64 electrodes (firing rate, Hz/ch; 1 s bins); therefore, an upward shift in the histogram indicates an increase in the firing frequency of the recorded DRG neuronal population. The TSC, the total number of spikes detected across all electrodes during the 10 min recording, is proportional to the area under the population firing-rate histogram and was used as an index of the magnitude of spike output. The SSR was defined as the fraction of spikes accompanied by at least one spike on another electrode within ±20 ms, that is, the proportion of firing that occurred nearly simultaneously at spatially separate sites. Because synchronized activity of DRG neurons has been observed in vivo in mouse models of neuropathic pain, and enhancing the synchrony rather than the overall level of DRG activity has been reported to induce pain-like behavior [19,20], the SSR was used as an index of population-level temporal coordination that is distinct from the amount of firing captured by the TSC.

**Figure 1:**
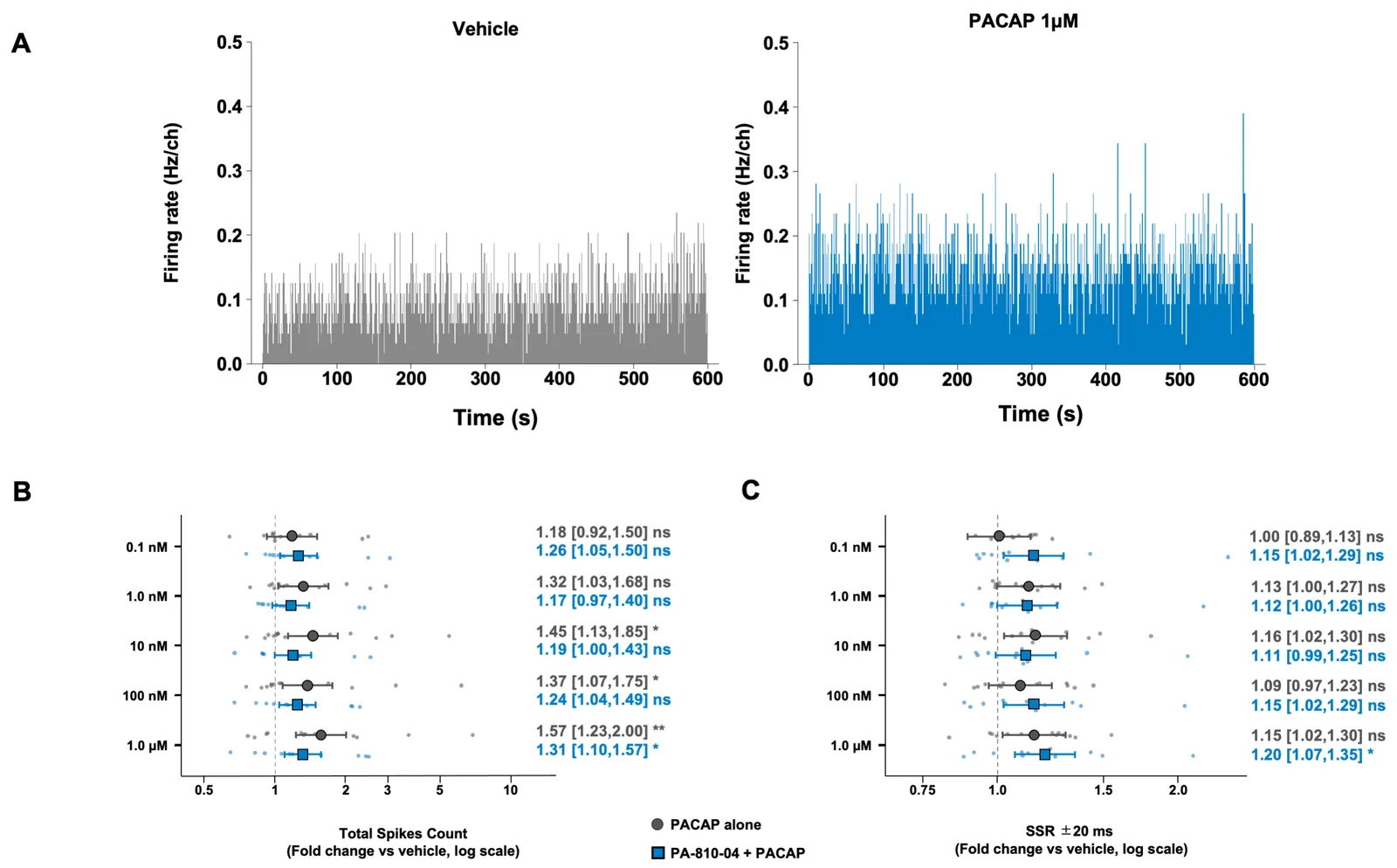
Exogenous PACAP enhances spontaneous and coordinated activity in cultured DRG neurons. (**A**) Representative population firing-rate histograms from cultured DRG neurons under vehicle and PACAP (1 µM) (10 min recordings; 64 electrodes). The *y*-axis shows the firing rate averaged across electrodes (Hz/ch; 1 s bins); an upward shift indicates increased firing of the DRG neuronal population. (**B**) Concentration–response relationship for total spike count (TSC) following exogenous PACAP application (0.1 nM–1 µM; *n* = 13 cultures). Data are shown as forest plots: symbols, fold-change point estimates (estimated marginal means, EMMs) versus vehicle (gray circles, PACAP alone; blue squares, PA-810-04 + PACAP, shown as paired rows for each concentration); horizontal bars, 95% confidence intervals (back-transformed from the log scale). Per-concentration significance (Holm-corrected): ns, not significant; * *p* < 0.05; ** *p* < 0.01. PACAP increased TSC concentration-dependently (likelihood-ratio test, *χ*^2^ = 15.74, *df* = 5, *p* = 0.008). With PA-810-04 pretreatment, the overall concentration-dependence was no longer significant (*n* = 11; *χ*^2^ = 12.11, *df* = 6, *p* = 0.060), with a significant increase remaining at 1 µM; the pretreatment × concentration interaction was not significant (*χ*^2^ = 4.42, *df* = 5, *p* = 0.49). (**C**) Concentration–response relationship for synchronous spike ratio (SSR; ±20 ms window). PACAP increased SSR concentration-dependently (likelihood-ratio test, *χ*^2^ = 11.11, *df* = 5, *p* = 0.049). With PA-810-04 pretreatment, the overall concentration-dependence remained significant (*n* = 11; *χ*^2^ = 14.12, *df* = 6, *p* = 0.028); the pretreatment × concentration interaction was not significant (*χ*^2^ = 5.60, *df* = 5, *p* = 0.35). Symbols are as described in (**B**).

To characterize the direct effect of PACAP on spontaneous DRG neuronal activity, cultured DRG neurons were exposed to PACAP (0.1 nM–1 µM; *n* = 13; Figure 1A: representative population firing-rate histogram). The magnitude of the spike output was examined as the first evaluation axis. In the representative firing-rate histogram, the population firing rate was higher under PACAP (1 µM) than under vehicle (Figure 1A), indicating increased firing of the DRG neuronal population. Consistent with this, PACAP increased the TSC in a concentration-dependent manner (likelihood ratio test [LRT], *χ*^2^ = 15.74, *df* = 5, *p* = 0.008; Figure 1B). Compared with the vehicle, the fold change was 1.18 at 0.1 nM (Holm *p* = 0.190), 1.32 at 1 nM (Holm *p* = 0.055), 1.45 at 10 nM (Holm *p* = 0.012), 1.37 at 100 nM (Holm *p* = 0.033), and 1.57 at 1 µM (Holm *p* = 0.002), demonstrating that the present MEA system reliably detects pharmacologically induced changes in DRG neuronal activity. Subsequently, we examined the second evaluation axis, coordinated firing, using the SSR. PACAP also increased the SSR (±20 ms window), although this effect was weaker than that on TSC (LRT, *χ*^2^ = 11.11, *df* = 5, *p* = 0.049; Figure 1C). The fold change relative to vehicle was 1.00 at 0.1 nM (95% confidence interval [CI]: 0.89–1.13; Holm *p* = 0.936), 1.13 at 1 nM (95% CI: 1.00–1.27; Holm *p* = 0.162), 1.16 at 10 nM (95% CI: 1.02–1.30; Holm *p* = 0.096), 1.09 at 100 nM (95% CI: 0.97–1.23; Holm *p* = 0.321), and 1.15 at 1 µM (95% CI: 1.02–1.30; Holm *p* = 0.096). To examine whether these responses were sensitive to PAC1 receptor antagonism, DRG neurons were pretreated with the PAC1 receptor antagonist PA-810-04 (10 µM) before PACAP application. In the presence of PA-810-04, the fold changes in TSC tended to be smaller at 1 nM–1 µM PACAP, and the overall concentration dependence was no longer statistically significant (*n* = 11; LRT, *χ*^2^ = 12.11, *df* = 6, *p* = 0.060; Figure 1B), although a significant increase remained at 1 µM after Holm correction. The SSR remained concentration-dependent in the presence of PA-810-04 (*n* = 11; LRT, *χ*^2^ = 14.12, *df* = 6, *p* = 0.028; Figure 1C). However, direct comparison between the PACAP-alone and PA-810-04 + PACAP groups revealed no significant pretreatment × concentration interaction for either TSC (*χ*^2^ = 4.42, *df* = 5, *p* = 0.49) or SSR (*χ*^2^ = 5.60, *df* = 5, *p* = 0.35). Moreover, because the PACAP-induced increase in SSR was itself small (maximum fold change, 1.16; no concentration reached significance after Holm correction), the present data were insufficient to determine whether PA-810-04 altered the SSR response. Therefore, exogenous PACAP directly enhanced spontaneous DRG neuronal activity, although the contribution of PAC1 receptor activation could not be established in this series. This series nonetheless served as a pharmacological positive control for the subsequent experiments.

### 2.3. PA-810-04 Attenuated L-OHP-Induced Hyperexcitability and Coordinated Firing of Cultured DRG Neurons

**Figure 2:**
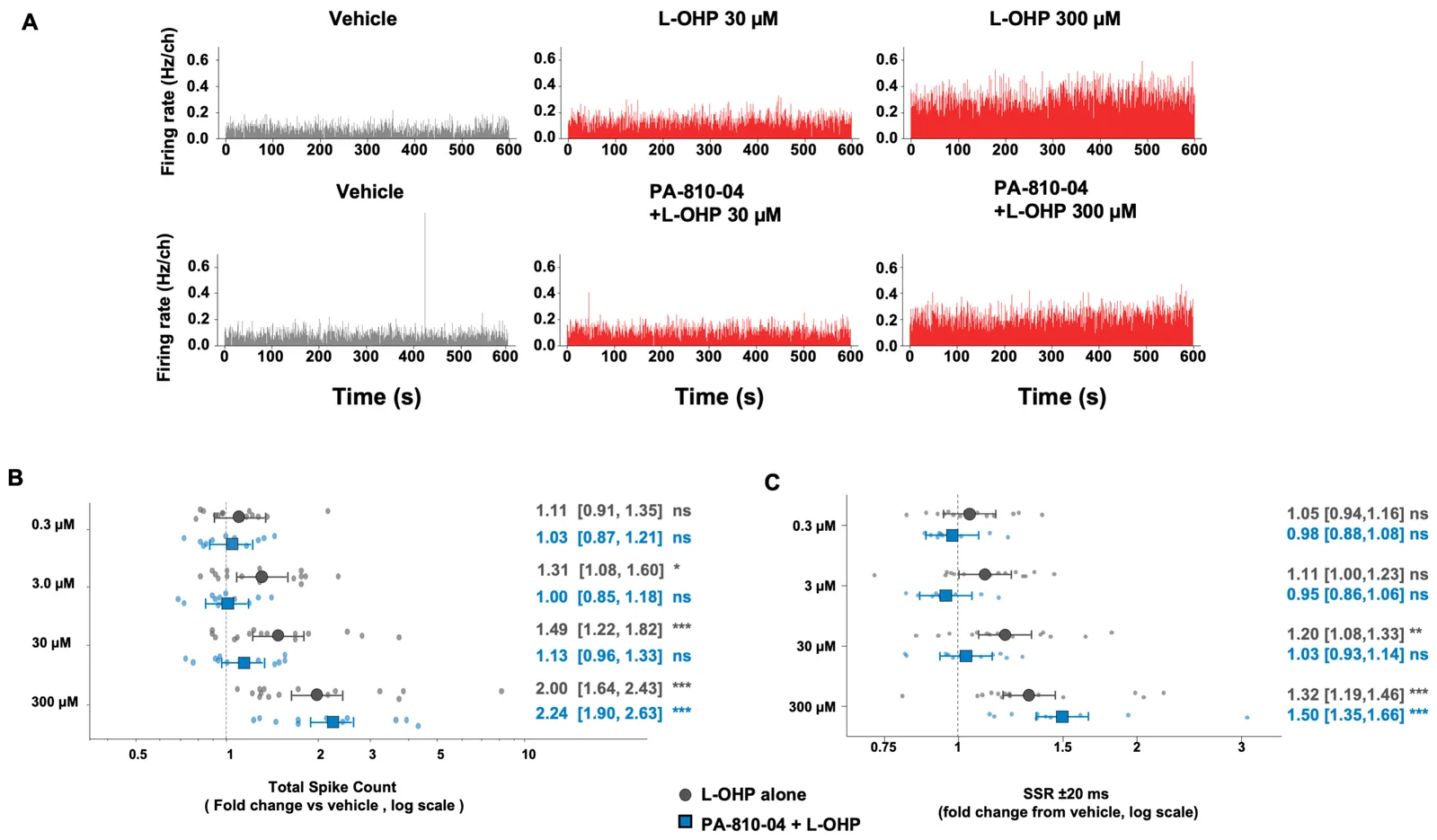
PA-810-04 attenuates L-OHP-induced hyperexcitability and coordinated firing of cultured DRG neurons. (**A**) Representative population firing-rate histograms recorded from a representative cultured DRG preparation under the indicated conditions (10-min recordings; 64 electrodes). The *y*-axis shows the firing rate averaged across electrodes (Hz/ch; 1-s bins). (**B**) Concentration-dependent changes in total spike count (TSC) following L-OHP exposure in the absence or presence of PA-810-04. Gray circles indicate the L-OHP-alone group (*n* = 15), and blue squares indicate the PA-810-04 + L-OHP group (*n* = 11). (**C**) Concentration-dependent changes in synchronous spike ratio (SSR) in the L-OHP-alone group (gray circles, *n* = 15) and the PA-810-04-pretreated group (blue squares, *n* = 11). SSR was calculated as the proportion of spikes with at least one coincident spike detected on another electrode within a ±20 ms time window. Data are presented as estimated marginal means (EMMs) ± 95% confidence intervals, expressed as fold changes relative to vehicle on a logarithmic scale, in both (**B**) and (**C**). Statistical significance was assessed by planned per-concentration comparisons with Holm correction. The pretreatment × concentration interaction was significant for both measures (likelihood-ratio test: TSC, *χ*^2^ = 12.2, *df* = 4, *p* = 0.016 (**B**); SSR, *χ*^2^ = 18.4, *df* = 4, *p* = 0.001 (**C**)). ns, not significant; * *p* < 0.05; ** *p* < 0.01; *** *p* < 0.001.

We evaluated the effect of L-OHP on the excitability of cultured DRG neurons (*n* = 15; Figure 2A; representative population firing-rate histogram). In the representative firing-rate histogram, the population firing rate was higher after L-OHP exposure than under vehicle (Figure 2A). Consistent with this, L-OHP exposure increased the TSC in a concentration-dependent manner (LRT, *χ*^2^ = 42.3, *df* = 4, *p* < 0.001; Figure 2B). Compared with the vehicle, the fold change reached 1.31 at 3 µM (Holm *p* = 0.014), 1.49 at 30 µM (Holm *p* < 0.001), and 2.00 at 300 µM (Holm *p* < 0.001), demonstrating that L-OHP enhances the spike output of DRG neurons in a dose-dependent manner.

The SSR (±20 ms window) also increased in a concentration-dependent manner under L-OHP exposure (*n* = 15; LRT, *χ*^2^ = 29.7, *df* = 4, *p* < 0.001; Figure 2C). The fold change relative to vehicle was 1.11 at 3 µM (95% CI: 1.00–1.23; Holm *p* = 0.090), 1.20 at 30 µM (95% CI: 1.08–1.33; Holm *p* = 0.001), and 1.32 at 300 µM (95% CI: 1.19–1.46; Holm *p* < 0.001). The significant increase at 30 µM or higher concentrations suggests that L-OHP increases not only the amount of firing but also the proportion of near-coincident firing across DRG neuronal populations. The primary analysis used a ±20 ms coincidence window, but a comparable concentration-dependent increase was confirmed using a wider ±100 ms window (Supplementary Figure S2).

To examine the effect of PA-810-04 (10 µM) pretreatment on L-OHP-induced increases in TSC, we compared the L-OHP-alone group (*n* = 15) with the PA-810-04 + L-OHP group (*n* = 11) (Figure 2B). The pretreatment × concentration interaction was significant (linear mixed-effects model [LMM] interaction LRT: *χ*^2^ = 12.2, *df* = 4, *p* = 0.016). The attenuating effect of PA-810-04 was apparent within the 0.3–30 µM L-OHP range but was no longer evident at the high concentration of 300 µM (Figure 2B, bottom row).

In the SSR comparison between the PA-810-04 + L-OHP group (*n* = 11) and the L-OHP-alone group (*n* = 15), the pretreatment × concentration interaction was significant (LMM interaction LRT, *χ*^2^ = 18.4, *df* = 4, *p* = 0.001; Figure 2C). In the per-concentration planned comparisons, the SSR was lower in the PA-810-04-pretreated group than in the L-OHP-alone group within the 3–30 µM L-OHP range tested, indicating attenuation of coordinated firing. In contrast, at the high concentration (300 µM), the SSR of the PA-810-04 + L-OHP group did not fall below that of the L-OHP-alone group, and no significant effect of PA-810-04 was observed.

Collectively, these results indicated that PA-810-04 pretreatment attenuated both the magnitude of excitability and population-level coordinated firing induced by L-OHP in DRG neurons within the L-OHP range examined in this study.

To assess whether the L-OHP-induced increase in SSR could be explained by simple proportional scaling with the firing rate, we evaluated the relationship between the fold change in TSC and the fold change in SSR for each recording (Supplementary Figure S3). The slope of the log–log relationship was 0.40 (*r*^2^ = 0.73, *p* < 0.001), which was well below 1.0. This indicates that SSR increased more gradually than the firing rate. However, because the SSR is a bounded proportion, a sublinear relationship between SSR and firing rate alone does not rule out a rate-dependent contribution to the coincidence measure.

### 2.4. PA-810-04 Attenuated Hyperexcitability and Coordinated Firing of Cultured DRG Neurons Induced by Conditioned Medium from SGC-Enriched Cultures

Previous studies have shown that SGCs modulate the excitability of adjacent DRG neurons through bidirectional neuron–glia signaling [21,22] and that L-OHP activates and functionally modifies SGCs in vitro [6]. Therefore, we examined whether soluble factors released from L-OHP-treated SGC-enriched cultures are sufficient to enhance DRG neuronal activity.

**Figure 3:**
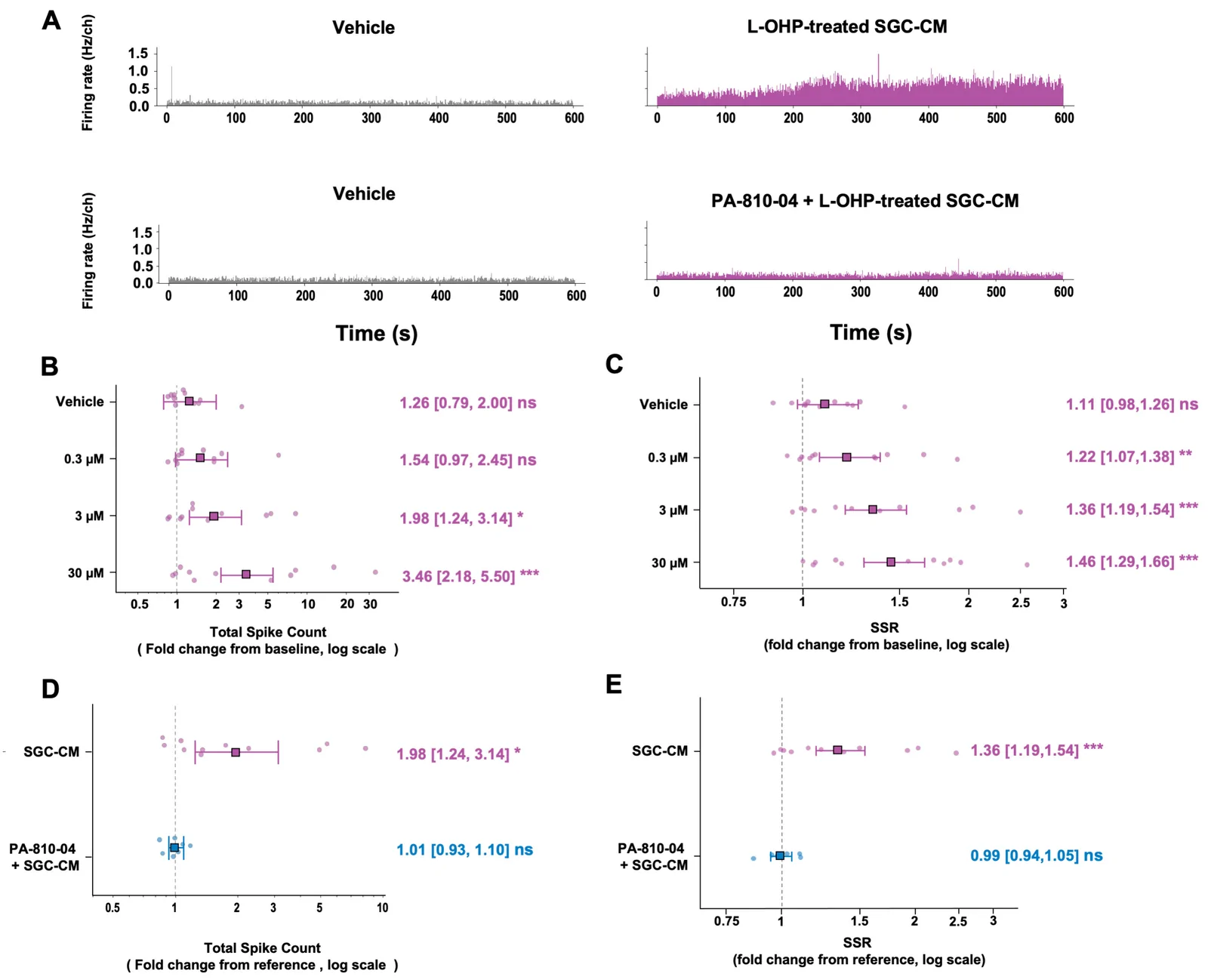
PA-810-04 attenuates DRG neuronal hyperexcitability and coordinated firing induced by conditioned medium from L-OHP-treated satellite glial cell (SGC)-enriched cultures. (**A**) Representative population firing-rate histograms recorded from a representative cultured DRG preparation under the indicated conditions (10 min recordings; 64 electrodes). The *y*-axis shows the firing rate averaged across electrodes (Hz/ch; 1 s bins). (**B**) Concentration-dependent changes in total spike count (TSC) following the application of conditioned medium (CM) collected from SGC-enriched cultures exposed to vehicle or L-OHP (0.3–30 µM) for 2 h (*n* = 11). Values are expressed as fold change relative to the baseline (pre-CM) recording. Purple squares indicate the SGC-CM group. (**C**) Concentration-dependent changes in SSR following application of CM collected from vehicle- or L-OHP-treated SGC-enriched cultures (*n* = 11). Values and symbols are as in (**B**). (**D**) Effect of PA-810-04 pretreatment (10 µM) on TSC induced by CM from SGC-enriched cultures exposed to 3 µM L-OHP. Purple squares indicate the SGC-CM-alone group (*n* = 11), and blue squares indicate the PA-810-04 + SGC-CM group (*n* = 7). Values are normalized to the corresponding pretreatment condition in each experimental group. Asterisks indicate significant differences from baseline (Holm-corrected planned comparisons). The between-group interaction was significant (likelihood-ratio test, *χ*^2^ = 4.77, *df* = 1, *p* = 0.029). (**E**) Effect of PA-810-04 pretreatment (10 µM) on SSR induced by CM from SGC-enriched cultures exposed to 3 µM L-OHP. Symbols and normalization are as in (**D**). The between-group interaction was significant (likelihood-ratio test, *χ*^2^ = 5.64, *df* = 1, *p* = 0.018). ns, not significant; * *p* < 0.05; ** *p* < 0.01; *** *p* < 0.001.

This conditioned medium (CM) from SGC-enriched cultures (hereafter SGC-CM), prepared from cultures exposed to L-OHP (0.3, 3, or 30 µM) for 2 h, was tested on DRG cultures (*n* = 11; Figure 3A: representative population firing-rate histogram; Figure 3B: concentration-dependent changes in TSC). In the representative firing-rate histogram, CM from SGC-enriched cultures exposed to 3 µM L-OHP raised the population firing rate relative to vehicle-CM (Figure 3A). Consistent with this, the L-OHP-exposed SGC-CM increased the TSC in a concentration-dependent manner (LRT, *χ*^2^ = 26.2, *df* = 4, *p* < 0.001). Relative to baseline, the fold change was 1.26 for the vehicle-CM (Holm *p* = 0.335) and increased to 1.98 (Holm *p* = 0.012) for the 3 µM L-OHP-CM and 3.46 (Holm *p* < 0.001) for the 30 µM L-OHP-CM. Therefore, soluble factors released from L-OHP-exposed SGC-enriched cultures can enhance the spike output of DRG neurons.

Subsequently, we examined whether SGC-CM altered coordinated firing using the SSR (±20 ms window; *n* = 11; Figure 3C). A significant increase relative to the baseline was observed beginning at 0.3 µM L-OHP treatment (LRT, *χ*^2^ = 32.8, *df* = 4, *p* < 0.001). The fold change in L-OHP-exposed SGC-CM relative to baseline was 1.22 at 0.3 µM (95% CI: 1.07–1.38; Holm *p* = 0.005), 1.36 at 3 µM (95% CI: 1.20–1.54; Holm *p* < 0.001), and 1.46 at 30 µM (95% CI: 1.29–1.66; Holm *p* < 0.001), all of which were significantly increased. Thus, soluble factors released from L-OHP-exposed SGC-enriched cultures enhance not only the excitability magnitude of DRG neurons but also the coordinated firing among these neurons. Notably, at the lowest concentration tested (0.3 µM), the SSR was significantly increased, whereas the corresponding increase in TSC did not reach significance (fold change 1.54; Figure 3B). Because the absence of a significant TSC increase does not establish that the firing rate was unchanged, a rate-dependent contribution to this response cannot be excluded, although the dissociation suggests that the SSR increase was not fully explained by a higher firing rate. The same log–log analysis applied to the SGC-CM condition yielded an even shallower slope of 0.28 (95% CI: 0.24–0.33; *r*^2^ = 0.79, *p* < 0.001; Supplementary Figure S4).

Because 3 µM was the lowest L-OHP-exposure concentration at which SGC-CM produced a significant increase in TSC, this condition was selected to test pharmacological blockade of PAC1 receptor (Figure 3A,D). When PA-810-04 (10 µM) was added to the DRG MEA culture before application of 3 µM L-OHP-exposed SGC-CM, TSC in the PA-810-04 + SGC-CM group (*n* = 7) remained comparable to that in the vehicle control (Fold change [FC] = 1.02, *p* = 0.79; Figure 3D) and, thus differed markedly from the SGC-CM-alone condition (*n* = 11; FC = 1.98). The between-group interaction analysis revealed a significant inhibitory effect of PA-810-04 pretreatment on the TSC (interaction LRT, *χ*^2^ = 4.77, *df* = 1, *p* = 0.029).

With PA-810-04 (10 µM) pretreatment, the SSR following application of 3 µM L-OHP-CM remained unchanged (FC = 1.00, *n* = 7; SGC-CM-alone FC = 1.36, *n* = 11). In the between-group interaction analysis, PA-810-04 pretreatment significantly inhibited the SSR (interaction LRT, *χ*^2^ = 5.64, *df* = 1, *p* = 0.018; Figure 3E).

### 2.5. Exploratory Analysis of the Temporal Irregularity of Population Firing (CV of ISI)

As an exploratory analysis, we evaluated the coefficient of variation of the inter-spike interval (CV of ISI) as an index of the temporal irregularity of array-wide population firing (Supplementary Figure S5). Neither direct L-OHP exposure nor exogenous PACAP produced significant concentration-dependent changes or pretreatment × concentration interactions (all interactions, *p* > 0.05). In contrast, L-OHP-exposed SGC-CM decreased the CV of ISI in a concentration-dependent manner (LRT, *χ*^2^ = 18.27, *df* = 4, *p* = 0.001), whereas PA-810-04 pretreatment had no significant effect (interaction *p* = 0.493). These findings indicate that, unlike TSC and SSR, the temporal irregularity of population firing was largely unaffected by PAC1 receptor antagonism under the present experimental conditions.

## 3. Discussion

### 3.1. Overview

In this study, we used MEA recordings from cultured rat DRG neurons to examine rapid L-OHP-induced changes in neuronal activity as an in vitro model of acute OIPN. The principal findings were as follows: (i) L-OHP increased both TSC, reflecting the overall spike output, and SSR, reflecting temporally coordinated firing across neuronal populations. (ii) Pretreatment with the PAC1 receptor antagonist attenuated L-OHP-induced changes in both readouts within a 0.3–30 µM concentration range. (iii) Conditioned medium prepared from L-OHP-exposed SGC-enriched cultures reproduced increases in both TSC and SSR in DRG neuron culture; and (iv) PAC1 receptor antagonist pretreatment also attenuated SGC-CM-mediated responses. To our knowledge, this is the first study to demonstrate that L-OHP-related DRG hyperexcitability involves not only increased spike output but also altered population-level temporal coordination and that soluble factor signaling from L-OHP-exposed SGC-enriched cultures affects both neuronal excitability and coordinated firing in a manner consistent with PACAP/PAC1 involvement. Thus, these findings identify a PAC1 receptor antagonist-sensitive component of early L-OHP-induced DRG hyperexcitability and suggest that PACAP/PAC1 receptor signaling may contribute to both direct neuronal responses and paracrine modulation by SGC-enriched cultures during the early phase of OIPN.

### 3.2. Methodological Significance of the Dual-Axis Analysis Using TSC and SSR

A central feature of the present study was the use of TSC and SSR as complementary readouts of DRG neuronal activity. The TSC reflects the magnitude of the spike output and therefore provides an index of overall excitability. In contrast, the SSR quantifies the temporal coordination of firing across neuronal populations. This distinction is important because an increase in spike output does not necessarily imply a proportional change in the temporal organization of neuronal activity. Thus, SSR enables the detection of a population-level coordinated firing phenotype that cannot be identified using TSC alone.

The relevance of this dual-axis approach is supported by our data. Direct L-OHP exposure increased the TSC (Figure 2B) and SSR (Figure 2C) in a concentration-dependent manner, indicating that L-OHP altered both the magnitude and temporal organization of DRG neuronal activity. More directly, CM prepared with the lowest L-OHP concentration (0.3 µM) significantly increased the SSR without a significant increase in the TSC (Figure 3B,C). Because a non-significant change in TSC does not establish that the firing rate was unchanged, this dissociation should be regarded as suggestive rather than conclusive. Nonetheless, it is consistent with the SSR capturing information that is not fully reflected in TSC.

Although this finding should be interpreted with caution, it further raises the possibility that coordinated firing may serve as a more sensitive functional biomarker than overall spike output for detecting early alterations in DRG neuronal function evoked by conditioned medium from SGC-enriched cultures. Therefore, the combined use of TSC and SSR allowed us to evaluate not only whether DRG neurons became hyperexcitable but also how their activity was organized at the population level in this cellular model relevant to acute OIPN.

The exploratory CV of the ISI analysis additionally distinguished cross-channel coincidence from the overall temporal irregularity of array-wide firing. Although the SSR consistently detected pharmacological modulation by PA-810-04, the array-wide CV of the ISI showed little change upon direct L-OHP exposure or PACAP stimulation. These observations suggest that PAC1 receptor antagonism preferentially influences the coincidence of firing across recording channels rather than the overall temporal irregularity of population firing, further supporting the complementary value of SSR as a functional readout of coordinated activity.

This is particularly relevant for acute OIPN. Clinically, acute OIPN can occur during L-OHP infusion or within hours after administration before overt axonal degeneration becomes apparent. This rapid time course suggests that early functional changes in sensory neuron activity may contribute to the initial phase of L-OHP-induced sensory disturbances. This MEA-based approach provides an in vitro framework for capturing early functional changes. By incorporating SSR, the model extends the conventional spike-output-based assessment and provides access to population-level temporal organization, a feature that may provide a useful framework for investigating early sensory neuron dysfunction in acute OIPN.

### 3.3. L-OHP-Related DRG Hyperexcitability and Attenuation by PAC1 Receptor Antagonist Pretreatment

L-OHP increased TSC in cultured DRG neurons in a concentration-dependent manner. This finding is consistent with previous studies showing that L-OHP enhances sensory neuron excitability through various mechanisms, including altered voltage-gated sodium (Na_v_), potassium (K_v_), and calcium (Ca_v_) channel functions; transient receptor potential (TRP) channel modulation; and mitochondrial dysfunction [4,23,24,25]. In the present study, L-OHP also increased the SSR, indicating that L-OHP affects the magnitude of DRG neuronal firing and temporal coordination of neuronal population activity.

PA-810-04 pretreatment attenuated both TSC and SSR responses to L-OHP within the 0.3–30 µM range. The pretreatment × concentration interaction was significant for both readouts, indicating that PA-810-04 modulated not only spike output but also coordinated firing in this in vitro DRG neuronal model. By contrast, in the exogenous PACAP series the pretreatment × concentration interaction was not significant for either readout (Figure 1B,C), and the PACAP-induced increase in SSR was too small to determine whether it was altered by PA-810-04. Whether the responses to bath-applied PACAP depend on PAC1 receptors therefore could not be established in the present study, and the relationship between these responses and the PA-810-04-sensitive components of the L-OHP- and SGC-CM-evoked responses remains to be determined.

At 300 µM L-OHP, the antagonistic effect was not observed, suggesting that high-concentration L-OHP responses may involve additional PAC1-independent mechanisms, such as direct ion-channel effects or cellular stress pathways. Because cytotoxicity and off-target effects were not directly assessed in the present study, these results should be interpreted with caution.

These findings are consistent with previous reports implicating PACAP/PAC1 signaling in neuropathic pain and L-OHP-induced pain behavior [9,10,11,12,13,14,15]. Although the present study did not directly quantify PACAP release or assess PAC1 receptor activation, the consistent pharmacological effects observed with both direct L-OHP exposure and SGC-CM treatment support the involvement of a PAC1 receptor antagonist-sensitive component. In addition, the use of PA-810-04 as a selective PAC1 receptor antagonist is based on its previously reported pharmacological characterization [13], and its receptor selectivity was not independently verified in the present preparations. Thus, our findings should be interpreted as supporting rather than conclusively proving the involvement of PACAP/PAC1 signaling.

### 3.4. Soluble-Factor Signaling from SGC-Enriched Cultures Amplifies PAC1 Receptor Antagonist-Sensitive DRG Hyperexcitability

SGCs form the immediate microenvironment surrounding DRG neurons and modulate sensory neuronal excitability under neuropathic conditions [5,6]. Previous studies have shown that L-OHP functionally modulates SGCs [6], and that SGC-derived secreted factors can enhance DRG neuronal excitability [26,27]. More recently, exosomes secreted by mouse SGCs treated with 4 µM L-OHP for 24 h were shown to be endocytosed by DRG neurons, where they upregulated ASIC3 and TRPV1 expression; intrathecal injection of these exosomes into naïve mice induced mechanical hypersensitivity [28]. In addition, a satellite glial GLRX3–HMGB1–TLR4 axis was identified as a driver of aging-biased chronic OIPN in aged mice. This mechanism was not engaged in young mice and was not detectable at acute stages [29]. These studies differ from the present work in their temporal profiles, proposed mechanisms, and outcome measures. In the exosome study, mechanical hypersensitivity developed over 24–72 h after injection and depended on vesicular cargo transfer [28], whereas the mechanism identified in aged mice involved a post-translational redox switch upstream of NF-κB-dependent transcription and operated over weeks to months [29]. Both studies primarily assessed changes in ion channel expression or pain-related behavior, whereas the SGC-CM responses examined here emerged within minutes of application and were assessed directly by measuring changes in neuronal electrical activity. Thus, our findings complement these previous studies rather than recapitulate their specific mechanisms and do not address aging-dependent chronicity of OIPN. Despite these differences, all three studies highlight the functional importance of the neuron–glia axis in neuropathic pain. The exosome study demonstrated vesicular transfer from SGCs to DRG neurons and its consequences for ion channel expression, whereas the aged-mouse study identified a specific redox-dependent signaling mechanism associated with chronic OIPN. The present findings extend this broader framework by demonstrating that SGC-CM can alter DRG neuronal firing within minutes of exposure, providing a high-temporal-resolution electrophysiological readout of neuron–glia communication. Based on these observations, we examined whether SGC-CM prepared after L-OHP exposure could alter DRG neuronal activity in the present MEA system.

One of the principal findings of the present study was that conditioned medium from L-OHP-exposed SGC-enriched cultures reproduced the major activity phenotypes observed after direct L-OHP exposure. Importantly, these effects were observed along the two activity axes. The increase in TSC indicates an enhanced spike output, whereas the increase in SSR indicates altered temporal coordination of DRG neuronal population activity. Thus, soluble-factor signaling from SGC-enriched cultures affects both the magnitude of DRG neuronal activity and population-level temporal organization.

Although the direct L-OHP and SGC-CM paradigms cannot be compared as simple concentration-equivalent exposures, the SGC-CM results suggest a leftward shift in the functional responsiveness of DRG neuronal activity. In particular, CM derived from SGC-enriched cultures exposed to the lowest L-OHP concentration significantly increased SSR, whereas TSC did not show a corresponding significant increase under these conditions. This pattern is consistent with the possibility that soluble factors released from L-OHP-exposed SGC-enriched cultures lower the effective response threshold for coordinated DRG neuronal activity. As residual L-OHP was not directly quantified or removed, this interpretation should be regarded as a working hypothesis that requires further validation.

**Figure 4:**
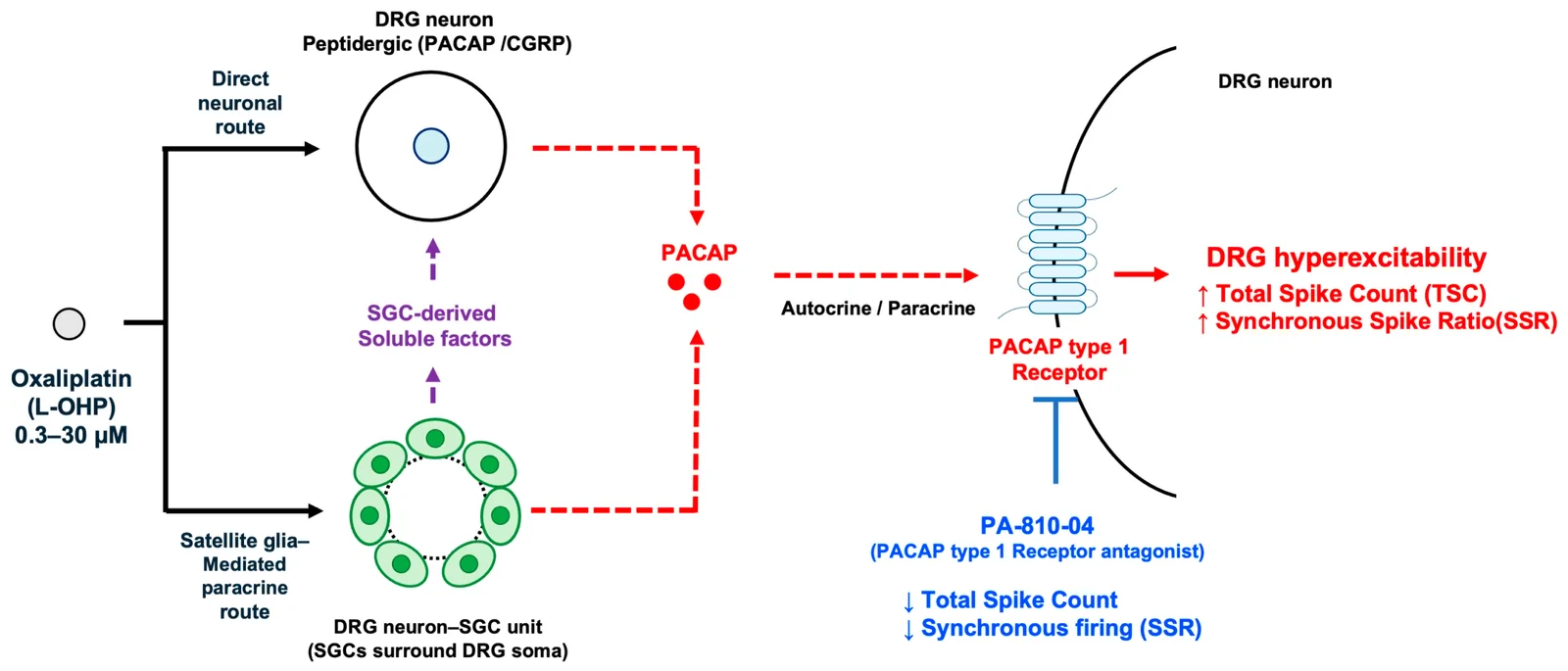
Proposed dual-route model for oxaliplatin (L-OHP)-induced DRG hyperexcitability and the PAC1 receptor antagonist-sensitive component (schematic). Oxaliplatin (L-OHP) is proposed to enhance dorsal root ganglion (DRG) neuronal excitability through two parallel pathways: a direct neuronal route involving ion channels, including Na_v_, K_v_, TRPA1, TRPM8, and Ca_v_ channels, and a paracrine route involving soluble factor(s) released from satellite glial cell (SGC)-enriched cultures. The factor(s) responsible were not identified in the present study. The SGC-enriched cultures used to prepare the conditioned medium were not characterized for identity or purity, and residual L-OHP was not removed before collection. Both routes increase neuronal excitability, as reflected by the increase in the total spike count (TSC; Figure 1B, Figure 2B and Figure 3B,D) and synchronous spike ratio (SSR; Figure 1C, Figure 2C and Figure 3C,E). Because both direct L-OHP exposure and L-OHP-exposed SGC-CM responses were attenuated by PA-810-04, both routes appear to include a PAC1 receptor antagonist-sensitive component. The direct neuronal route may potentially involve autocrine signaling, whereas the second route is consistent with a paracrine mechanism. However, PACAP release and PAC1 receptor activation were not directly measured. Therefore, the role of endogenous PACAP/PAC1 signaling and convergence of the two routes on this pathway remain hypothetical. Solid arrows indicate relationships supported by the present findings and/or previous studies, whereas dashed arrows denote hypothetical mechanisms that remain to be experimentally validated.

Importantly, pretreatment with the PAC1 receptor antagonist attenuated the SGC-CM-induced increase in both TSC and SSR. This indicates that a PAC1 receptor antagonist-sensitive component is present in the paracrine responses evoked by conditioned medium from SGC-enriched cultures as well as in direct neuronal responses to L-OHP. Although the soluble mediator(s) responsible for SGC-CM-induced effects were not identified, these observations support a model in which a PAC1 receptor antagonist-sensitive mechanism constitutes a shared component through which both neuronal and glial pathways contribute to early DRG hyperexcitability (Figure 4).

### 3.5. Proposed Two-Component Model Relevant to Acute OIPN

Acute OIPN develops during or within a few hours of L-OHP administration and is characterized by cold-evoked hyperesthesia and dysesthesia. Most previous mechanistic studies have focused on the direct effects of L-OHP on DRG neurons, including altered Na_v_ channel function [23], sensitization of TRPA1 and TRPM8 channels [24,30], and potential contribution of Ca_v_ channels [31]. Without dismissing the conventional ion channel dysfunction model, our data support a two-component conceptual framework for L-OHP-related DRG hyperexcitability relevant to acute OIPN in this cellular model (Figure 4). As summarized schematically in Figure 4, the first component is the direct neuronal response to L-OHP. In this component, L-OHP acts on DRG neurons and increases spike output, as reflected by TSC, while also enhancing coordinated firing, as reflected by SSR. This component is consistent with previous studies showing that L-OHP can alter sensory neuron excitability through direct effects on ion channels, including the Na_v_, K_v_, TRPA1, TRPM8, and Ca_v_ channels [2,4].

The second component, newly highlighted in the present study, involves soluble factor signaling from SGC-enriched cultures. In this context, L-OHP-exposed SGC-enriched cultures release soluble mediators that enhance DRG neuronal activity. The present SGC-CM experiments indicate that this response affects both TSC and SSR, suggesting that these soluble factors can modulate both the magnitude and temporal coordination of sensory neuron population activity. The attenuation of this response by PAC1 receptor antagonist pretreatment further suggests that this soluble factor response is consistent with PACAP/PAC1 involvement. Because the identity and purity of the SGC-enriched cultures were not characterized, residual L-OHP was not removed, and the mediators responsible for the observed effects were not identified, this second component should be regarded as a working hypothesis rather than an established mechanism.

This framework may help explain why sensory symptoms emerge rapidly before overt structural degeneration develops. The present study did not directly reproduce clinically acute OIPN because cold sensitivity, pain behavior, and patient symptoms were not assessed. Nevertheless, by examining the rapid changes in DRG neuronal activity after short-term L-OHP exposure, the present MEA system provides a cellular model relevant to acute OIPN. Within this model, dual-axis analysis allows the evaluation of early L-OHP-related functional changes as not only increased spike output but also altered temporal coordination of DRG neuronal population activity.

### 3.6. Limitations and Future Perspectives

This study had some limitations. First, cultured rat DRG neurons and conditioned media prepared from SGC-enriched cultures were used in this in vitro study. Therefore, these results cannot be directly extrapolated to acute clinical OIPN. Future studies using patient-derived systems are required to determine the translational relevance of the present findings.

Second, the cultures used for the CM preparation were not immunocytochemically characterized. Cell identity was not confirmed using SGC markers, and culture purity was not quantified. Therefore, contamination by neurons, Schwann cells, or fibroblasts cannot be excluded, and the observed CM effects cannot be exclusively attributed to SGCs. Accordingly, we refer to these preparations as SGC-enriched cultures. The isolation protocol on which our preparation was based has been reported to yield cultures in which 98–99% of the cells are positive for the glial markers GFAP, S100, Kir4.1 and glutamine synthetase [6]. This finding suggests that SGCs may also predominate in our cultures. However, because our protocol was modified and purity was not re-examined here, this expectation is based on the previously reported protocol rather than on direct verification. Furthermore, interrogation of a harmonized cross-species single-cell reference atlas of DRG and trigeminal ganglion [32] revealed that *Adcyap1* expression is not restricted to SGCs, and that multiple non-neuronal cell populations are present in these ganglia. Thus, if represented in our preparations, other cell types, including Schwann cells, pericytes, endothelial cells, fibroblasts, and immune cells, could potentially contribute to the observed CM effects. Future studies should combine marker-based cell identification and the quantitative assessment of SGC purity, together with depletion or enrichment of candidate non-neuronal populations, to establish the cellular origin of the active factors.

Third, the molecular mediators responsible for the effects of SGC-CM were not identified. Although attenuation by PAC1 receptor antagonist pretreatment was consistent with PACAP/PAC1 involvement, PACAP release, PAC1 activation, and secretion of other soluble mediators from the SGC-enriched cultures were not directly measured. Future studies should directly quantify PACAP and other candidate soluble mediators, including cytokines, in SGC-CM, assess PAC1 activation in DRG neurons, and test whether antagonists of candidate mediators modify TSC and SSR responses.

Fourth, residual L-OHP in SGC-CM may have contributed to the observed effects. As SGC-CM was applied without L-OHP removal after exposure of the SGC-enriched cultures, residual L-OHP could not be completely excluded. However, because the nominal residual L-OHP concentration after dilution was substantially lower than the concentrations that produced detectable effects under direct L-OHP application, the effects of SGC-CM are unlikely to be explained solely by residual L-OHP. Nevertheless, future studies should include an L-OHP washout before CM collection, direct quantification of residual L-OHP, and concentration-matched residual L-OHP controls to validate our findings. In addition, because the SGC-CM concentration series (vehicle-CM, 0.3, 3, and 30 µM L-OHP-CM) was applied cumulatively to the same culture, order and carry-over effects on the concentration-dependent SGC-CM responses cannot be fully excluded. A randomized-order or single-concentration design would be required to address this.

Fifth, the present study did not systematically analyze direct DRG–SGC co-culture interactions. SGC-CM experiments were designed to isolate soluble factor signaling. However, they did not address whether direct SGC–neuron contact further modifies L-OHP-related DRG hyperexcitability, for example, through gap junction coupling or close-apposition (e.g., purinergic) signaling between SGCs and neurons [5]. Future studies using predefined DRG–SGC co-culture protocols are required to distinguish soluble factor-mediated effects from contact-dependent SGC–neuron interactions.

Sixth, the cellular site of PAC1 receptor expression was not resolved. Although *Adcyap1r1* mRNA was detected by RT-PCR in the neuron-enriched DRG cultures, these preparations also contain non-neuronal cells, precluding the assignment of this signal specifically to neurons. In addition, PAC1 receptor protein expression was not examined. Interrogation of a harmonized cross-species single-cell reference atlas of DRG and trigeminal ganglion [32] revealed limited detection of *Adcyap1r1* transcripts among the rat DRG neurons represented in the dataset. However, rat cells constitute only a small fraction of that atlas, and single-cell and single-nucleus transcriptomic methods may incompletely detect low-abundance transcripts. Thus, limited detection does not establish an absence of expression. Furthermore, the recordings in the present study were obtained from cultures prepared at embryonic day 14, a developmental stage not represented in that resource. Accordingly, the PA-810-04-sensitive responses reported here cannot be localized to neuronal PAC1 receptors, and the cellular site at which PACAP/PAC1 receptor signaling operates in this system remains unresolved. L-OHP-induced chemical injury may also induce or upregulate PAC1 receptor expression, which would reconcile the present pharmacological findings with the low baseline *Adcyap1r1* levels reported in naïve tissue. Nevertheless, the attenuation of both the direct L-OHP- and the SGC-CM-evoked responses by PA-810-04 is consistent with the involvement of PACAP/PAC1 receptor signaling in this system. Addressing these questions will require cell type-resolved approaches before and after L-OHP exposure, such as PAC1 receptor immunocytochemistry or in situ hybridization combined with neuronal and glial markers.

Finally, the SSR should be interpreted as a population-level coordinated firing readout rather than a direct measure of pain or clinical sensory symptoms. Although synchronized DRG neuronal activity has been implicated in neuropathic pain processing [19,20] and enhanced afferent input can drive central sensitization [33], the present SSR findings do not establish a direct causal link to pain behavior. In addition, the mechanism underlying the coordinated firing detected here remains to be determined. Because DRG neurons are not thought to form conventional synaptic connections with one another in vivo, the increase in SSR may partly reflect rate-dependent coincidences, network interactions established in culture, or SGC-mediated coupling.

In vivo voltage imaging of DRG with the positively tuned genetically encoded indicator ASAP4.4-Kv has recently demonstrated cell-to-cell electrical synchronization between adjacent sensory neurons. This phenomenon was rarely observed in naïve mice, increased after peripheral inflammation or nerve injury, and was significantly reduced by the gap junction blocker carbenoxolone [34]. At the phenomenological level, these findings are consistent with the present results: coordinated firing increased relative to control in both systems, after peripheral inflammation or nerve injury in vivo and after short-term L-OHP exposure or SGC-CM application in our study. The two approaches, however, are subject to different potential confounders. In vivo optical recordings require the exclusion of respiratory- and heartbeat-related rhythmic signals, as addressed in the previous study [34], whereas extracellular recordings in culture are not subject to such motion-related artifacts. However, the latter cannot establish which neurons contributed to a given coincident event or whether those neurons were adjacent. Thus, cross-channel coincidence in culture should not be equated with synchronized neuronal activity in vivo. Nevertheless, the sensitivity of the in vivo phenomenon to gap junction blockade provides a specific experimental hypothesis to test in the present system. Subsequent work by the same group identified vasomotion in small blood vessels within injured ganglia as a trigger of clustered neuronal firing in vivo. This phenomenon was sensed by Piezo2 in sensory neurons with injured axons and was suppressed by anti-VEGF treatment [35]. This vascular mechanism depends on an intact vascular microenvironment, including smooth muscle cells and pericytes, as well as the spatial proximity of injured neurons to a shared vessel. These features are not preserved in dissociated cultures and therefore cannot readily explain the coincident firing observed in our MEA recordings. Which of the candidate mechanisms outlined above, if any, accounts for this activity remains to be determined. Neither study established whether gap junction-dependent synchronization [34] and vasomotion-driven neuronal firing [35] share a common mechanism. Moreover, the contribution of gap junctional coupling to the vascular mechanism has not been established.

Further analyses, including surrogate-based assessments of coincident firing and pharmacological interventions targeting glutamatergic transmission or gap junctions, will be required to clarify the mechanisms underlying the coincident activity observed in our system.

In conclusion, the present study identified a PAC1 receptor antagonist-sensitive component of early oxaliplatin-induced DRG hyperexcitability and suggested that this component is involved in both direct neuronal responses and paracrine signaling from SGC-enriched cultures. By combining MEA recordings with complementary analyses of the TSC and SSR, this study established a functional cellular model that captured early electrophysiological changes relevant to acute OIPN. These findings provide a mechanistic framework that supports further investigation of PA-810-04-sensitive mechanisms and the potential involvement of PACAP/PAC1 signaling in neuron–glia interactions in chemotherapy-induced peripheral neuropathy.

## 4. Materials and Methods

### 4.1. Animals and Ethical Approval

Primary dorsal root ganglion (DRG) cultures were prepared from Wistar rat embryos on embryonic day 14 (E14). Timed pregnant Wistar rats were purchased from Japan SLC (Shizuoka, Japan). All animal procedures were approved by the Animal Experimentation Committee of Kagoshima University (approval no. MD23057; approved on 8 November 2023) and was carried out in accordance with the Regulations for Animal Experimentation of Kagoshima University, the Fundamental Guidelines for Proper Conduct of Animal Experiments and Related Activities in Academic Research Institutions (Ministry of Education, Culture, Sports, Science, and Technology, Japan), and the Standards Relating to the Care and Management of Laboratory Animals and Relief of Pain (Notification No. 88, 2006, Ministry of the Environment, Japan).

### 4.2. DRG Explant Preparation and Culture

DRG neurons were isolated from Wistar rat embryos at E14 and plated as explants on multielectrode array (MEA) dishes coated with poly-D-lysine (25 µg/mL; Sigma-Aldrich, St. Louis, MO, USA). Spontaneous activity recordings from cultured DRG neurons using MEA constitute a well-established in vitro system for stable assessment of neuronal activity [16,17]. The culture medium consisted of Neurobasal medium (Gibco, Waltham, MA, USA) supplemented with 2% B-27 supplement (Gibco), 1% GlutaMAX (Gibco), nerve growth factor (NGF 2.5S, 100 ng/mL; Alomone Labs, Jerusalem, Israel), and penicillin/streptomycin (100 U/mL and 100 µg/mL, respectively; Gibco). Fetal bovine serum (FBS; Gibco) was added at 10% only on days in vitro (DIV) 0. From DIV 1 onward, FBS was omitted, and uridine (17.5 µg/mL; Amresco, Solon, OH, USA) together with 5-fluoro-2′-deoxyuridine (7.5 µg/mL; Sigma-Aldrich, St. Louis, MO, USA) was added to suppress the proliferation of non-neuronal cells. Half of the medium was replaced every 3 days, and the cultures were maintained at 37 °C in a humidified atmosphere of 5% CO_2_ in air. Electrophysiological recordings and pharmacological experiments were performed on DRG cultures at 10–14 DIV.

### 4.3. Satellite Glial Cell-Enriched Culture and Conditioned Medium Preparation

Satellite glial cell (SGC)-enriched cultures were prepared from the same DRG explant cultures used for the MEA recordings, following a modified version of the method described by Schmitt et al. [6]. DRG explants were cultured for 14 days, after which the cells were enzymatically dissociated with 0.25% trypsin-EDTA (Gibco) for 15 min at 37 °C. The dissociated cells were counted and seeded onto poly D-lysine-coated (25 µg/mL) 6-well plates at a density of 10,000 cells per well. The SGC-enriched cultures were maintained in the same Neurobasal/B27-based medium used for the DRG cultures and allowed to adhere and proliferate for 72 h. For preparing the conditioned medium (CM), SGC-enriched cultures were exposed to oxaliplatin (L-OHP) (0.3, 3, or 30 µM) or vehicle (5% glucose solution; Otsuka Pharmaceutical Factory, Naruto, Japan) for 2 h at 37 °C. After exposure, the culture supernatants were collected. Each supernatant was added to the DRG MEA culture at a final 1:100 dilution in recording medium. Under this final dilution, the nominal residual L-OHP concentration in the CM corresponds to one-hundredth of the concentration applied to the SGC-enriched cultures, that is, 3, 30, and 300 nM for CM derived from 0.3, 3, and 30 µM L-OHP exposure, respectively. For SGC-CM evaluation, a 10 min baseline recording was obtained before CM application, after which vehicle-CM, 0.3 µM L-OHP-CM, 3 µM L-OHP-CM, and 30 µM L-OHP-CM were applied cumulatively. After each application, the culture was allowed to equilibrate for 5 min, and then recorded for 10 min. To examine whether the novel organic small-molecule antagonist of the PAC1 receptor, PA-810-04 [13] (see Section 4.6), attenuated SGC-CM-induced hyperexcitability, a separate group of DRG MEA cultures was pretreated with PA-810-04 (10 µM) for 30 min before applying 3 µM L-OHP-CM. In this protocol, vehicle, PA-810-04, and SGC-CM recordings were performed sequentially for 10 min.

### 4.4. RT-PCR and Immunocytochemistry for PACAP and PAC1 Receptor

To confirm that the primary DRG cultures used in the present study expressed components of the PACAP/PAC1 signaling pathway, the expression of PACAP and the PAC1 receptor was examined before the electrophysiological experiments.

PACAP (*Adcyap1*) mRNA expression was evaluated by reverse transcription polymerase chain reaction (RT-PCR), and PACAP peptide expression was confirmed by immunocytochemistry using a PACAP-specific antibody. PAC1 receptor (*Adcyap1r1*) mRNA expression was examined by RT-PCR. These analyses were performed using neuron-enriched DRG cultures prepared under the same conditions as those used for the MEA experiments.

Total RNA was isolated from cultured DRG preparations using a NucleoSpin RNA kit (TaKaRa Bio, Shiga, Japan) according to the manufacturer’s instructions. First-strand cDNA was synthesized from the extracted total RNA using the PrimeScript™ II 1st strand cDNA Synthesis Kit (TaKaRa Bio, Shiga, Japan) according to the manufacturer’s instructions. PCR amplification was performed using EmeraldAmp^®^ MAX PCR Master Mix (TaKaRa Bio, Shiga, Japan) with gene-specific primers for *Adcyap1* and *Adcyap1r1*. The PCR products were analyzed by agarose gel electrophoresis. The following gene-specific primers were used for RT-PCR: *Adcyap1*, 5′-cctttactacccagccgaca-3′ (forward) and 5′-ctgtctgtgaagatgccgtc-3′ (reverse); *Adcyap1r1*, 5′-cccactcttcggaatccact-3′ (forward) and 5′-taacggttcaccttccagct-3′ (reverse).

For immunocytochemistry, cultures were fixed with 4% paraformaldehyde (Merck, Darmstadt, Germany), permeabilized with 0.01% Triton X-100 (Nacalai Tesque, Kyoto, Japan), blocked with 1% bovine serum albumin (Nacalai Tesque, Kyoto, Japan), and incubated with an anti-PACAP-38 antibody (1:1000, Rabbit polyclonal, No. T-4473, BMA Biomedicals, Augst, Switzerland) and/or anti-CGRP antibody (1:1000, sheep polyclonal, Enzo Life Sciences, Farmingdale, NY, USA) overnight at room temperature, followed by incubation with an Alexa Fluor-conjugated secondary antibody (Abcam, Cambridge, UK) and DAPI (Dojindo, Kumamoto, Japan). Images were acquired using a confocal laser-scanning microscope (LSM 700; Carl Zeiss Microscopy, Jena, Germany). The representative results are shown in Supplementary Figure S1B.

### 4.5. Multielectrode Array Recordings

Extracellular recordings were performed using the MED64 system (Alpha MED Scientific, Osaka, Japan) as previously described. MEA recordings simultaneously capture spontaneous firing and pharmacological responses across multiple electrodes, allowing the quantification of temporal coordination within neuronal populations. The MEA dish contained 64 electrodes arranged in an 8 × 8 grid (electrode size, 50 × 50 µm; inter-electrode spacing, 150 µm). Signals were acquired at a sampling rate of 20 kHz. Recordings were conducted at 37 °C in a chamber maintained under 5% CO_2_ and 95% air. Each recording was performed for 10 min.

### 4.6. Drug Application

All drugs were freshly prepared on the day of the experiment and diluted in culture medium immediately before use. After the application of a drug or CM to the MEA dish, the culture was allowed to equilibrate for 5 min at 37 °C before initiating a 10 min MEA recording. Oxaliplatin (L-OHP) (TCI, Tokyo, Japan) was dissolved in a 5% glucose solution and applied at 0.3, 3, 30, or 300 µM. The 0.3–30 µM range encompasses concentrations commonly used in previous in vitro OIPN studies [6,23,30] and is consistent with the clinically relevant plasma concentration range of L-OHP [36]. PACAP-38 (Peptide Institute, Osaka, Japan) was dissolved in distilled water and applied at 0.1, 1, 10, 100 nM, or 1 µM. The PAC1 receptor antagonist PA-810-04, the structural name of which is 2-Amino-5-(3-trifluoromethoxyphenyl)-5,8-dihydropyrido [2,3-d]pyrimidine-4,7(3H,6H)-dione and is structurally identical to the compound ‘2o,’ a derivative from the mother compound PA-8 [13]. PA-810-04 was custom synthesized by Shionogi Pharma Co., Ltd. (Osaka, Japan) according to the previously reported synthetic procedure [13], was dissolved in dimethyl sulfoxide (DMSO; FUJIFILM Wako Pure Chemical Corporation, Osaka, Japan) and applied at 10 µM with a 30 min pretreatment. PA-810-04 was applied at 10 µM based on prior in vitro studies and published pharmacological characterization [13]. The final DMSO concentration in the bath did not exceed 0.1%. Each MEA dish was used in only one experimental protocol and was not reused.

### 4.7. Spike Detection and Activity Analysis

Raw waveforms from each electrode were band-pass filtered between 200 and 3000 Hz to isolate spike activity. Spikes were detected using the Spike Detector module of the MED64 system, with the detection threshold for each electrode set to ±5 × median(|signal|). A median-based threshold is commonly used as a spike-detection method that is robust to interchannel noise differences [18].

To visualize how population firing changed over time, a population firing-rate histogram was constructed. Spikes from all 64 electrodes were pooled and counted in consecutive 1 s bins, and each bin was divided by the bin width and by the number of electrodes:

*r*(*k*) = *n*(*k*)/(*N* × Δ*t*)

where *n*(*k*) is the number of spikes detected on all electrodes within bin *k*, *N* = 64 is the number of electrodes, and Δ*t* = 1 s is the bin width. The resulting value *r*(*k*) is expressed in Hz per channel (Hz/ch) and represents the mean firing rate of a single electrode during that bin. For example, *r*(*k*) = 0.3 Hz per channel corresponds to 0.3 spikes per second on an average electrode, that is, 19.2 spikes per second summed over the array. An increase in *r*(*k*) therefore indicates an increase in the firing of the recorded neuronal population.

The total spike count (TSC) was defined as the sum of all spikes detected across all 64 electrodes during the 10 min recording. The synchronous spike ratio (SSR) was defined as a coincidence-based measure of population firing coordination across recording channels. For each detected spike, a spike was classified as synchronous when at least one spike occurred on another recording channel within a ±20 ms temporal window. The SSR was calculated as the number of synchronous spikes divided by the total number of detected spikes. A ±20 ms window was used for the primary analysis because it is longer than the duration of an individual extracellular spike waveform yet short enough to capture near-coincident firing across channels. Because coincidence-based synchrony estimates may depend on the temporal window, sensitivity analyses were additionally performed using ±10, ±50, and ±100 ms. The direction and statistical significance of the primary findings were preserved across all window sizes (Supplementary Figure S2). To quantify the temporal irregularity of population firing, the coefficient of variation of the inter-spike interval (CV of ISI) was calculated as an exploratory index. For each recording, spikes detected on all 64 electrodes were pooled into a single array-wide spike train and sorted in time. The ISIs of this pooled train were calculated (zero-length intervals arising from simultaneous spikes were excluded), and the CV of the ISI was obtained as the standard deviation of these ISIs divided by their mean. This array-wide CV of the ISI was used as an index of the temporal irregularity of population firing for each recording condition. The CV of the ISI was computed from the same recordings and under the same quality-control criteria as the primary TSC and SSR analyses, and the results for all experimental series are presented in Supplementary Figure S5.

### 4.8. Experimental Design

The experimental groups were L-OHP alone (*n* = 15), PACAP alone (*n* = 13), PA-810-04 pretreatment + L-OHP (*n* = 11), PA-810-04 pretreatment + PACAP (*n* = 11), SGC-CM (*n* = 11), and PA-810-04 pretreatment + SGC-CM (*n* = 7). Each series was conducted independently. Each culture preparation was considered an independent biological sample (*n* units). The samples were prepared individually from separate dissections and culture sessions and did not represent repeated measurements of the same culture.

### 4.9. Statistical Analysis

Statistical analyses were performed using linear mixed-effects models (LMMs) to accommodate the repeated measures structure of the data. For each experimental series, the overall effect of concentration or condition was evaluated using likelihood ratio tests (LRTs). Per-concentration comparisons against the corresponding vehicle or baseline condition were pre-specified as planned comparisons and were performed for all series regardless of the significance of the overall effect, with multiple comparisons corrected using the Holm–Bonferroni method. To assess the influence of PA-810-04 pretreatment, between-group comparisons were performed using a model that included the pretreatment × concentration interaction, thereby examining whether PA-810-04 altered the concentration–response relationship of L-OHP. The SGC-CM experiments were performed similarly, and the difference between the PA-810-04-pretreated and untreated groups was evaluated using the interaction term. For the between-group comparison of SGC-CM, the pre-drug vehicle condition of each group was used as a reference. Specifically, the baseline (pre-CM; containing the L-OHP vehicle, glucose) was used as the reference for the SGC-CM alone group, whereas the vehicle condition (pre-PA, containing both the PA-810-04 vehicle, DMSO, and the L-OHP vehicle, glucose) was used as the reference for the PA-810-04 pretreatment group so that each group was normalized to its pretreatment state for comparison. For quality control, baseline outliers were excluded using a median absolute deviation applied uniformly to the TSC, CV of the ISI, and coordinated firing index SSR. The TSC and SSR were defined as the primary analysis indices, whereas the CV of the ISI was treated as an exploratory analysis. The results are reported as fold-change (FC) or model-derived estimates. Note that the FCs presented in the main text and figures are, in principle, LMM-derived estimates (the exponentiated form of log-scale coefficients equivalent to geometric means) rather than the arithmetic mean paired ratios of raw data. The vehicle-CM, L-OHP-CM (A3 dose–response), and PA-810-04 SGC-CM (B3 between-group comparison) analyses used the same within-recording baseline (pre-CM) as the reference level. The level of statistical significance was set at *p* < 0.05. The LMM used log-transformed response variables [log (*y* + 1) for count metrics and log (*y* + 0.1) for SSR], with condition as the fixed effect and recording as a random intercept. Models were fitted using the Powell optimizer, with restricted maximum likelihood (REML) used for point estimates and maximum likelihood (ML) re-estimation for model comparison using LRT.

For the primary linear mixed models, model adequacy was confirmed using log-scale residuals. Q–Q plots, the Shapiro–Wilk test, and residual-versus-fitted plots indicated mild departures from normality in the count-based metrics (TSC) and SSR of the L-OHP-alone and PACAP series (Shapiro–Wilk *p* < 0.001–0.045), driven by some outliers, whereas no appreciable departure was observed for the SGC-CM SSR (*p* = 0.224). No systematic heteroscedasticity was detected in the residual-versus-fitted plots of any model (Supplementary Figure S6). Satterthwaite-corrected degrees-of-freedom analyses yielded significance conclusions identical to those obtained by the Wald approach (results are presented as likelihood-ratio *χ*^2^ statistics and corresponding *p* values in the main text). Sensitivity analysis using leave-one-out (LOO) resampling, that is, repeated reanalysis after excluding each sample (Supplementary Table S1), also broadly maintained the major interaction findings. The only exception was the PA-810-04 × L-OHP total spike count interaction, where *p* rose to 0.068 in one LOO iteration (*p* < 0.05 in the remaining 25/26 iterations). The other primary interaction analyses (B1 SSR, B3 TSC, and B3 SSR) remained significant in all LOO iterations.

### 4.10. Software and the Use of Generative AI

Analyses were performed in Python 3.13.1 (NumPy 2.3.0, SciPy 1.15.3, pandas 2.3.0, statsmodels 0.14.6), and Satterthwaite-corrected degrees-of-freedom checks were performed using R version 4.0.1 with packages lme4 1.1.28 and lmerTest 3.1.3. A large language model (Claude, Anthropic; versions Claude Opus 4.7, Opus 4.8, Opus 5, and Opus 5.5, used via Claude Code between 2026 and the time of submission) was used to draft the code for the statistical analyses. All numerical statistics were computed using these software packages and were independently reproduced by the authors. Generative AI was not involved in the interpretation of the results or formulation of scientific conclusions.

## Data Availability

The data supporting the findings of this study are available from the corresponding author upon reasonable request.

## Supplementary Materials

The following supporting information can be downloaded at: https://www.mdpi.com/article/10.3390/ijms27219665/s1.

## Abbreviations

The following abbreviations are used in this manuscript:

OIPN	Oxaliplatin-induced peripheral neuropathy
DRG	Dorsal root ganglion
PACAP	Pituitary adenylate cyclase-activating polypeptide
PAC1	PACAP type 1 receptor
MEA	Multi-electrode array
TSC	Total spike count
SSR	Synchronous spike ratio
L-OHP	Oxaliplatin
SGCs	Satellite glial cells
CM	Conditioned medium
FC	Fold change
LRT	Likelihood ratio test
