# Supplementary material for: Selective PAC1 Receptor Antagonist, PA-810-04 Attenuates Oxaliplatin-Induced Hyperexcitability and Coordinated Firing in Cultured Dorsal Root Ganglion Neurons
Source: Int J Mol Sci. 2026 Oct 28;27(21):9665. doi: 10.3390/ijms27219665 (PMC13762488; doi:10.3390/ijms27219665)
Supplement: Supplementary file 1 [file ijms-27-09665-s001.zip › ijms-4641115-supplementary.pdf]

Article

Selective PAC1 Receptor Antagonist, PA-810-04 Attenuates Oxaliplatin-Induced Hyperexcitability and Coordinated Firing in Cultured Dorsal Root Ganglion Neurons

Taiyo Arima [1,2], Kazuhiro Takahama [2], Kimitaka Suetsugu [1,2], Yukiko Kobae [1], Haruki Iwai [3], Ichiro Takasaki [4], Takashi Kurihara [5] and Hideyuki Terazono [1,2,*]

[1] Department of Clinical Pharmacy and Pharmacology, Graduate School of Medical and Dental Sciences, Kagoshima University, 8-35-1 Sakuragaoka, Kagoshima 890-8544, Japan; ta126245@m.kufm.kagoshima-u.ac.jp (T.A.); ksuetsugu@kufm.kagoshima-u.ac.jp (K.S.); ykobae@m.kufm.kagoshima-u.ac.jp (Y.K.)

[2] Department of Pharmacy, Kagoshima University Hospital, 8-35-1 Sakuragaoka, Kagoshima 890-8520, Japan; takahama@kufm.kagoshima-u.ac.jp

[3] Department of Oral Anatomy and Cell Biology, Graduate School of Medical and Dental Sciences, Kagoshima University, 8-35-1 Sakuragaoka, Kagoshima 890-8544, Japan; haruki@dent.kagoshima-u.ac.jp

[4] Laboratory of Pharmacology, Graduate School of Pharma-Medical Sciences, University of Toyama, 3190 Gofuku, Toyama 930-8555, Japan; takasaki@eng.u-toyama.ac.jp

[5] Department of Pharmacology, Graduate School of Medical and Dental Sciences, Kagoshima University, 8-35-1 Sakuragaoka, Kagoshima 890-8544, Japan; tkmphm10@m.kufm.kagoshima-u.ac.jp

* Correspondence: terazono@m.kufm.kagoshima-u.ac.jp; Tel: +81-99-275-5543

Supplementary Materials

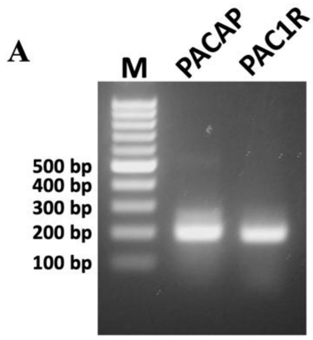

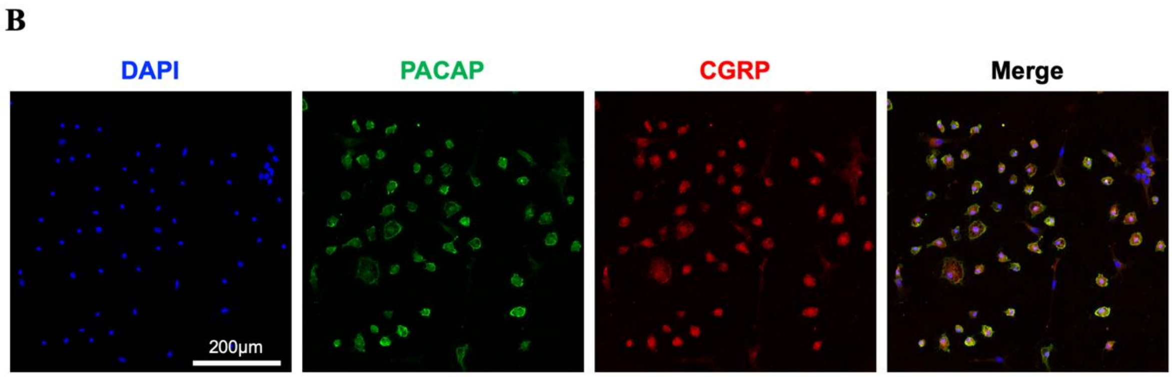

Figure S1. PACAP and PAC1 receptor expression in the primary culture of DRG. (A) Representative qualitative PCR detection of PACAP and PAC1 receptor (PAC1R). M: DNA size marker. (B) Representative confocal laser scanning microscopy images of cultured DRG neurons. DAPI (blue), PACAP (green), CGRP (red), and the merged image. Scale bar, 200 μm.

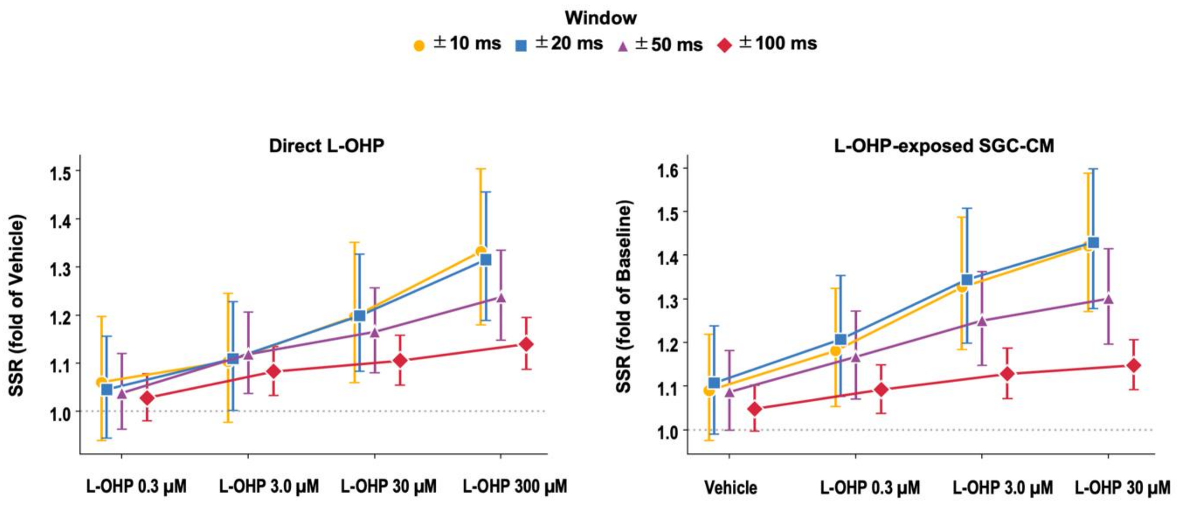

Figure S2. Sensitivity analysis of the synchronous spike ratio (SSR) using different synchrony windows (±10, ±20, ±50, and ±100 ms). SSR was recalculated using coincidence windows of ±10, ±20 (primary analysis), ±50, and ±100 ms for both the direct L-OHP treatment and L-OHP-exposed conditioned medium from SGC-enriched cultures (SGC-CM) treatment groups. Across all synchrony windows, L-OHP-induced increases in SSR were consistently observed. Although the magnitude of the relative increase was modestly reduced with wider coincidence windows, the concentration-dependent trend and the overall conclusions remained unchanged across all analysis conditions, supporting the robustness of the SSR analysis.

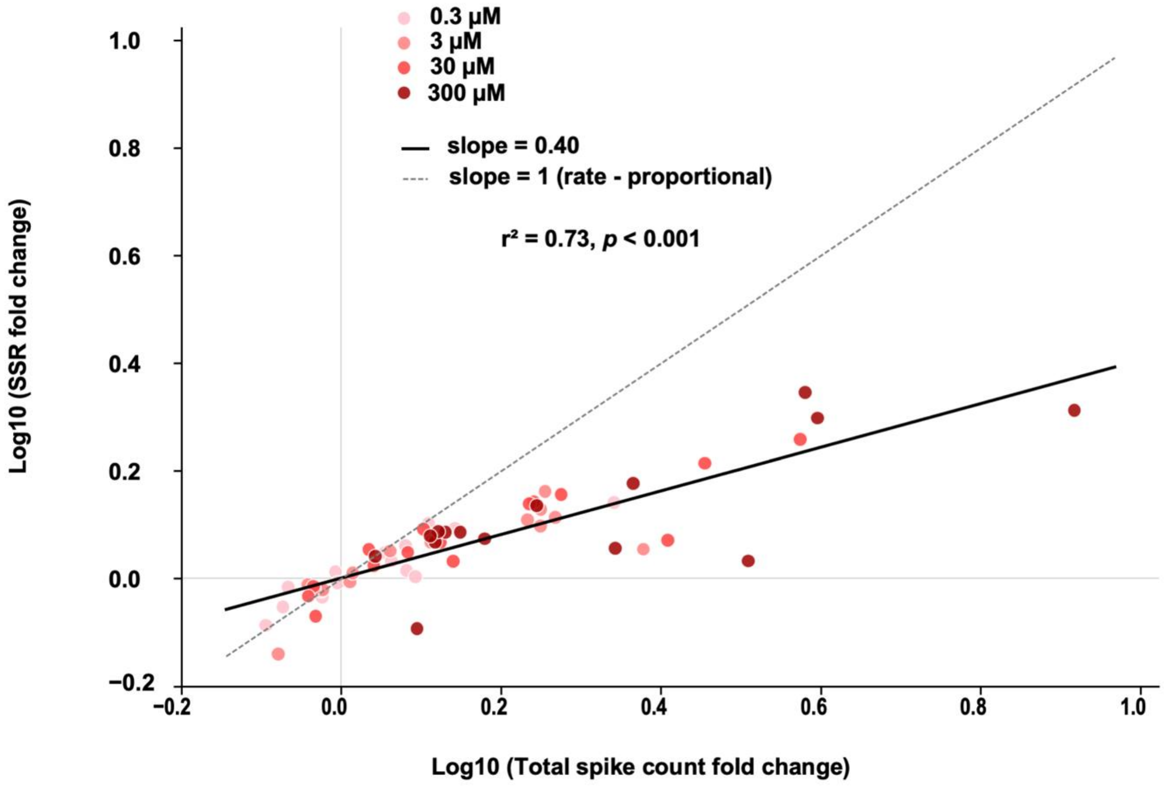

Figure S3. Relationship between total spike count (TSC) and synchronous spike ratio (SSR) fold changes following direct L-OHP application. Each point represents one L-OHP concentration in an individual recording (60 observations from 15 recordings). Fold changes in TSC (x-axis) and SSR (y-axis) were plotted in log–log space and analyzed by linear regression. The regression yielded a slope

of 0.40 (r^2 = 0.73, $p < 0.001$), substantially lower than the unity (rate-proportional) slope of 1.0, indicating that increases in SSR scaled sublinearly relative to increases in TSC.

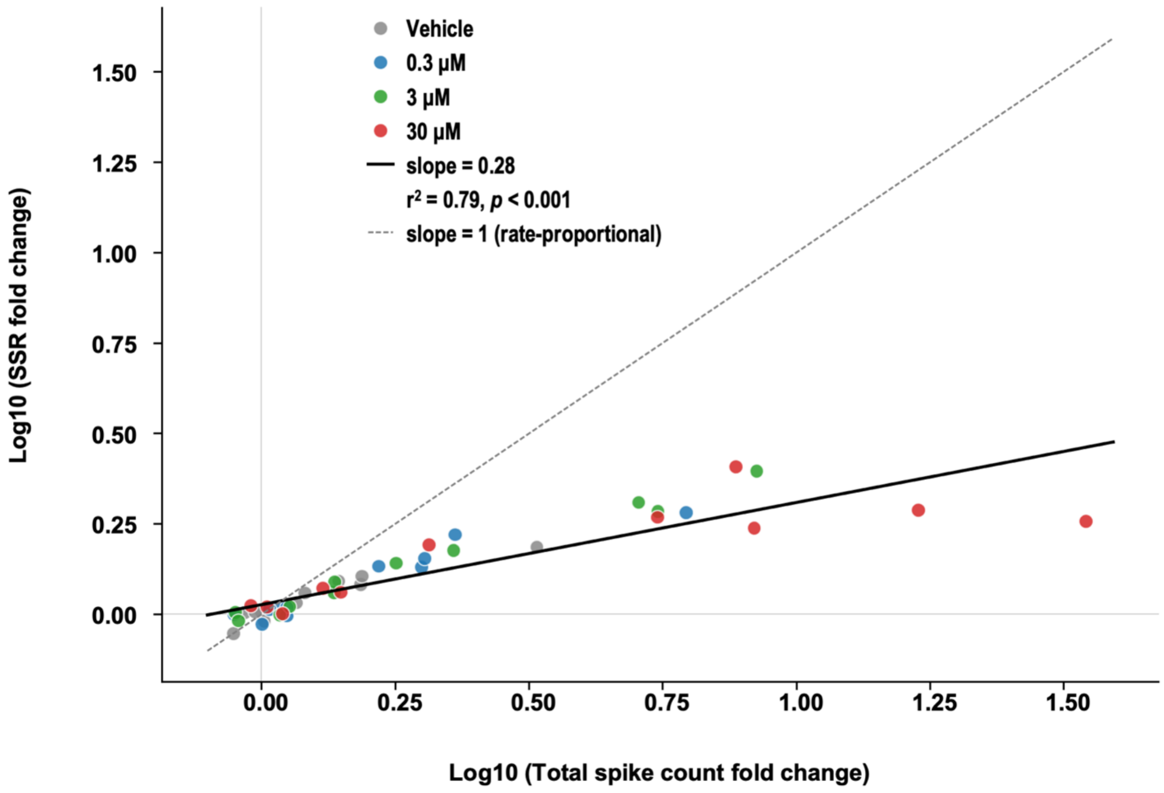

Figure S4. Relationship between total spike count (TSC) and synchronous spike ratio (SSR) fold changes following treatment with L-OHP-exposed conditioned medium from SGC-enriched cultures (SGC-CM). Fold changes in TSC (x-axis) and SSR (y-axis) were plotted in log–log space and analyzed by linear regression using 44 condition-level observations derived from 11 recordings (Vehicle and L-OHP-exposed SGC-CM prepared with 0.3, 3, or 30 μM L-OHP) after quality control. The estimated regression slope was 0.28 (95% CI, 0.237–0.328; r^2 = 0.79; $p < 0.001$), indicating a markedly sublinear relationship between the two measures. This relationship was more sublinear than that observed following direct L-OHP application (estimated slope = 0.40; Supplementary Figure S3).

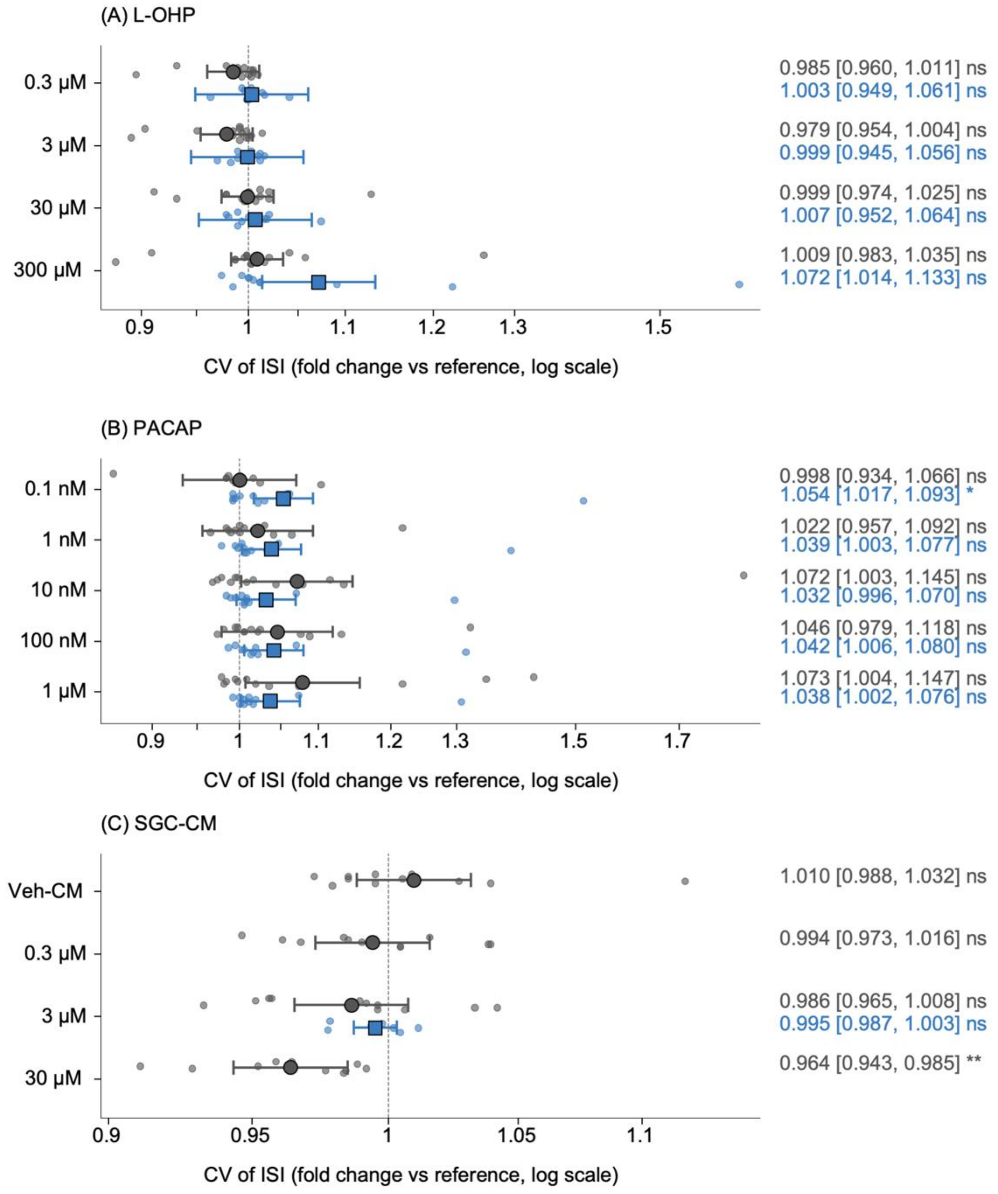

Figure S5. Coefficient of variation of the interspike interval (CV of ISI) as an exploratory index of the temporal irregularity of array-wide population firing. For each recording, the CV of ISI was calculated from the ISIs of a single spike train obtained by pooling the spikes of all 64 electrodes (array-wide CV of ISI), using the same recordings and quality-control criteria as the primary total spike count (TSC) and synchronous spike ratio (SSR) analyses. Data are shown as forest plots of fold changes relative to the corresponding reference condition: symbols, point estimates (estimated marginal means, EMMs) (gray circles, L-OHP, PACAP, or SGC-CM alone; blue squares, PA-810-04-pretreated groups); horizontal bars, 95% confidence intervals (back-transformed from the log scale). (A) Direct L-OHP exposure: L-OHP alone (gray circles; n = 15; likelihood-ratio test, χ^2 = 7.02, df = 4, p = 0.135) and PA-810-04 + L-OHP (blue squares; n = 11; χ^2 = 11.22, df = 5, p = 0.047), with no significant pretreatment × concentration interaction (χ^2 = 5.72, df = 4, p = 0.221). (B) Exogenous PACAP: PACAP alone (gray circles; n = 13; χ^2 = 9.41, df = 5, p = 0.094) and PA-810-04 + PACAP (blue squares; n = 11; χ^2 = 11.36, df = 6, p = 0.078), with no significant interaction (χ^2 = 7.56, df = 5, p = 0.182). (C) L-OHP-exposed conditioned medium from SGC-enriched cultures: SGC-CM alone (gray circles; vehicle-CM and CM prepared with 0.3, 3, or 30 μM L-OHP; n = 11) and PA-810-04 + SGC-CM (blue square; CM prepared with 3 μM L-OHP only; n = 7): the CV of ISI decreased in a concentration-dependent manner (χ^2 = 18.27, df = 4, p = 0.001), reaching significance for CM prepared with 30 μM

L-OHP (fold change 0.964; 95% CI, 0.943–0.985), whereas the interaction with PA-810-04 pretreatment was not significant (χ^2 = 0.47, *df* = 1, *p* = 0.493). Per-concentration significance (Holm-corrected): ns, not significant; * $p < 0.05$; ** $p < 0.01$. Because the pretreatment × concentration interaction was not significant in any series, the concentration effects shown for individual arms should not be interpreted as evidence of a difference between arms. The CV of ISI was analyzed as an exploratory measure, and *p*-values were not corrected across outcome measures.

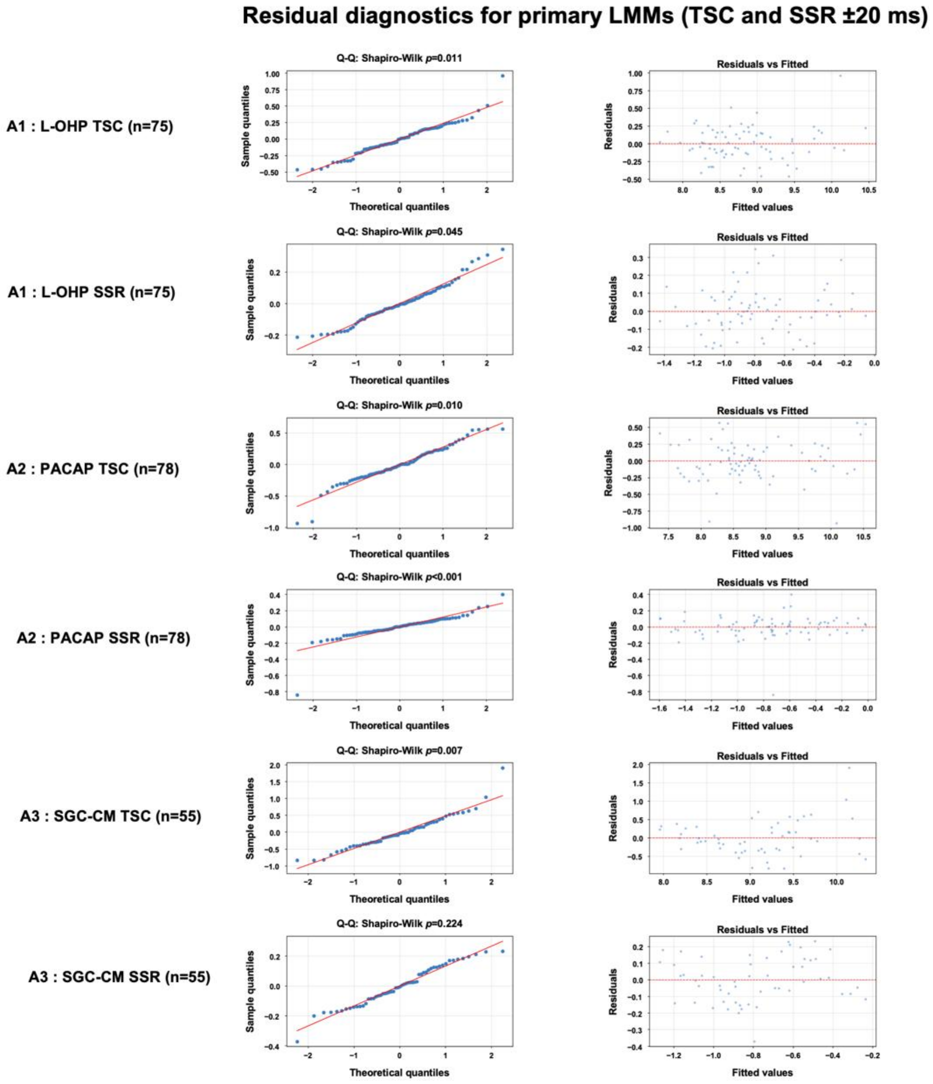

Figure S6. Residual diagnostics for the primary linear mixed-effects models. Model diagnostics for the six primary linear mixed-effects models (LMMs) fitted to log-transformed response variables. For each model, the left panel shows a normal quantile–quantile (Q–Q) plot of the residuals with the corresponding Shapiro–Wilk normality test *p*-value, and the right panel shows residuals versus fitted values. Each row corresponds to one model: A1, L-OHP total spike count (TSC) (*n* = 75; *p* = 0.011); A1, L-OHP synchronous spike ratio (SSR) (*n* = 75; *p* = 0.045); A2, PACAP TSC (*n* = 78; *p* = 0.010); A2, PACAP SSR (*n* = 78; *p* < 0.001); A3, SGC-CM TSC (*n* = 55; *p* = 0.007); and A3, SGC-CM SSR (*n* = 55; *p* = 0.224). SSR was calculated using a ±20 ms synchrony window. Mild departures from normality were observed for several models, particularly those based on count-derived endpoints, whereas the residual-versus-fitted plots showed no evidence of substantial heteroscedasticity. The observed deviations were attributable to a small number of outlying observations and were further evaluated by leave-one-out sensitivity analysis (Supplementary Table S1) and an alternative Satterthwaite analysis, both of which yielded consistent conclusions.

Table S1. Leave-one-out (LOO) sensitivity analysis of primary interaction effects. Leave-one-out resampling was performed for the two between-group comparisons (B1: PA-810-04 + L-OHP vs. L-OHP alone; B3: PA-810-04 + SGC-CM vs. SGC-CM alone), each evaluated for the two primary outcome measures (TSC and SSR ±20 ms; four interaction analyses in total). The PACAP series (B2) was not included because its pretreatment × concentration interactions were not significant (TSC, x^2 = 4.42, *df* = 5, *p* = 0.49; SSR, x^2 = 5.60, *df* = 5, *p* = 0.35), and the LOO procedure was applied only to significant interaction effects. For each analysis, each recording was omitted once, the linear mixed-effects model was refitted, and the interaction likelihood ratio test (LRT) was repeated. The table summarizes the number of iterations, range and median of *p*-values, number of statistically significant interactions ($p < 0.05$), and any influential recordings whose omission resulted in $p \geq 0.05$. Only one LOO iteration for the B1 total spike count (TSC) interaction yielded a non-significant result (p = 0.068), whereas all other interaction analyses remained statistically significant throughout the LOO procedure.

Analysis	N (LOO iterations)	*p* min	*p* max	*p* median	Significant (*p* < 0.05)	Influential obs (*p* ≥ 0.05)
B1, TSC	26	0.001577	0.06753	0.01851	25/26	One culture in the PA-810-04 + L-OHP group (*p* = 0.068)
B1, SSR	26	0.0001939	0.005164	0.001332	26/26	None
B3, TSC	18	0.01568	0.04996	0.03874	18/18	None
B3, SSR	18	0.008815	0.03737	0.02321	18/18	None
